# Nanoporous and nano thickness film-forming bioactive composition for biomedical applications

**DOI:** 10.1038/s41598-022-12280-8

**Published:** 2022-05-17

**Authors:** Naga Thirumalesh Chevala, Lalit Kumar, Vimal Veetilvalappil, Aranjani Jesil Mathew, Bemma Paonam, Ganesh Mohan, Shamee Shastry, Krishnan Balasubramanian, C. Mallikarjuna Rao

**Affiliations:** 1 Department of Pharmaceutics, Manipal College of Pharmaceutical Sciences, Manipal Academy of Higher Education, Manipal, Karnataka 576104 India; 2 Department of Biotechnology, Manipal College of Pharmaceutical Sciences, Manipal Academy of Higher Education, Manipal, Karnataka 576104 India; 3 Department of Pharmacology, Manipal College of Pharmaceutical Sciences, Manipal Academy of Higher Education, Manipal, Karnataka 576104 India; 4 Department of Immunohematology and Blood Transfusion, Kasturba Medical College, Manipal Academy of Higher Education, Manipal, Karnataka 576104 India; 5 Department of Mechanical Engineering, Indian Institute of Technology, Madras, India

**Keywords:** Health care, Medical research, Materials science, Nanoscience and technology

## Abstract

Unmanageable bleeding is one of the significant causes of mortality. Attaining rapid hemostasis ensures subject survivability as a first aid during combats, road accidents, surgeries that reduce mortality. Nanoporous fibers reinforced composite scaffold (NFRCS) developed by a simple hemostatic film-forming composition (HFFC) (as a continuous phase) can trigger and intensify hemostasis. NFRCS developed was based on the dragonfly wing structure's structural design. Dragonfly wing structure consists of cross-veins and longitudinal wing veins inter-connected with wing membrane to maintain the microstructural integrity. The HFFC uniformly surface coats the fibers with nano thickness film and interconnects the randomly distributed cotton gauge (Ct) (dispersed phase), resulting in the formation of a nanoporous structure. Integrating continuous and dispersed phases reduce the product cost by ten times that of marketed products. The modified NFRCS (tampon or wrist band) can be used for various biomedical applications. The in vivo studies conclude that the developed Cp NFRCS triggers and intensifies the coagulation process at the application site. The NFRCS could regulate the microenvironment and act at the cellular level due to its nanoporous structure, which resulted in better wound healing in the excision wound model.

## Introduction

Unmanageable bleeding during combats, intraoperative, and accidental conditions can cause significant life threats to the causalities^1^. These conditions further lead to the total increase in peripheral vascular resistance, which causes hemorrhagic shock. Proper measures to control bleeding during and after the surgery is considered as a potential life threat^2,3^. Great blood vessels with injury lead to colossal blood loss, resulting in ≤ 50% lethality in combats and 31% in intraoperative conditions^1^. Heavy blood loss results in a decrease in total body volume, which reduces cardiac output. The rise in total peripheral vascular resistance and progressive impaired microcirculation leads to hypoxia of life-supporting organs. As the situation continues without effective intervention may lead to hemorrhagic shock^1,4,5^. Other complications include a progression of hypothermia and metabolic acidosis with impaired blood coagulation, making the blood coagulation process more difficult. The severe hemorrhagic shock is a higher risk of mortality^6–8^. For Class III (progressive stage) shocks, blood transfusion is necessary for the patient survival during intraoperative, postoperative morbidity, and mortality^8^. To overcome all the above-mentioned life-threatening conditions, we developed a nanoporous fibers reinforced composite scaffold (NFRCS) using a combination of water-soluble styptic polymers by utilizing minimal polymer concentration (0.5%).

**Figure 1:**
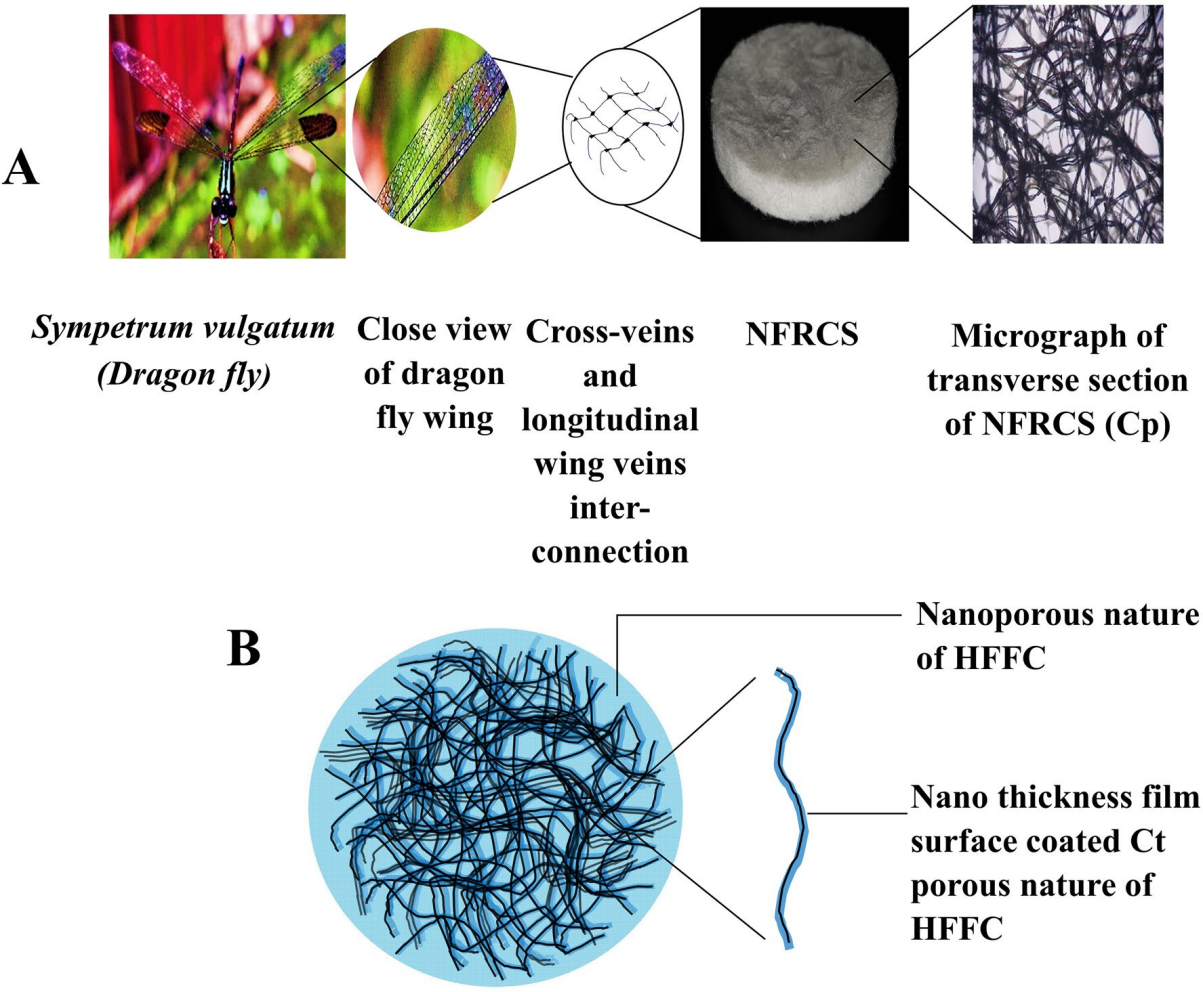
The image represents the schematic representation of NFRCS structural design based on dragonfly wing structure, (**A**). The image shows the comparative analogy of the dragonfly wing structure (cross vein and longitudinal wing veins interconnection) and micrograph of a transverse section of Cp NFRCS, (**B**). Schematic representation of NFRCS.

Using fiber reinforcement applications, economically viable products can be developed. The randomly arranged fibers resemble the dragonfly's wing structure, balanced with cross-veins and longitudinal wing veins. The cross-veins and longitudinal wing veins are inter-connected with the wing membrane (Fig. 1). NFRCS consists of a reinforced Ct as a skeletal system for better physical and mechanical strength (Fig. 1). The surgeons prefer the cotton gauge (Ct) during surgeries and dressing due to the affordable price and proficiency. Hence, considering its multiple benefits, including > 90% crystalline cellulose (imparts in the enhancement of hemostatic activity), Ct was used as a skeletal system of NFRCS^9,10^. The Ct was surface coated (nano thickness film formation was observed) and interconnected with the hemostatic film-forming composition (HFFC). The HFFC act as a matrix glue that holds the randomly arranged Ct together in shape. The developed design transfers stress within dispersed phases (reinforced fibers). By utilizing minimum polymer concentrations, attaining a nanoporous structure with good mechanical strength is challenging. Furthermore, it is not easy to customize in various shapes for different biomedical applications.

NFRCS was developed using HFFC as a continuous phase to sort out the above constraints. The HFFC is composed of different film-forming styptic polymers, which include chitosan (as a base styptic polymer) in combination with methylcellulose (MC), hydroxypropyl methylcellulose (HPMC 50 cp), and polyvinyl alcohol (PVA) (125 kDa), respectively as supportive polymers which facilitate to intensify the clot formation. The addition of polyvinyl pyrrolidine K30 (PVP K30) improves the moisture uptake ability of the NFRCS. Polyethylene glycol 400 (PEG 400) was added to improve polymer cross-linking within the hybrid polymeric blend. Three different HFFC hemostatic compositions (Cm HFFC, Ch HFFC, and Cp HFFC), i.e., chitosan with MC (Cm), chitosan with HPMC (Ch), and chitosan with PVA (Cp) were surface coated on the Ct. The hemostatic and wound healing activities of NFRCS were confirmed by various in vitro and in vivo characterization studies. The NFRCS suggested composite may be utilized to customize various scaffold forms to meet specific needs.

Furthermore, NFRCS can be modified into a bandage or roll form to cover the entire area of lower limb injuries and other body parts. Especially for combat limb injuries, the developed NFRCS design can be altered into half-arm or leg selves (Supplementary Figure S11). NFRCS can be developed into a wrist band with the help of tissue adhesive glue that can be employed to control the bleeding during serious suicidal serious wrist injuries. Our primary concern was to develop an NFRCS with a minimum polymer utilization that can be made available for a large population (below the poverty line) and can be accommodated in the first aid kit. The NFRCS design was simple, efficient, and economical, which is helpful for the natives and could make an impact globally.

## Materials and methods

Chitosan (molecular weight 80 kDa) and amaranth were purchased from Merck chemicals, India. Hydroxypropyl methylcellulose 50 Cp, polyethylene glycol 400, and methylcellulose were purchased from Loba Chemie Pvt. Ltd., Mumbai. Polyvinyl alcohol (molecular weight 125 kDa) (87–90% hydrolyzed) was purchased from National chemicals, Gujarat. Polyvinyl pyrrolidine K30 was purchased from Molychem, Mumbai; the sterilized cotton gauge was purchased from Ramaraju surgical cotton mills Ltd., Tamil Nadu, and Milli Q (Direct-Q3 Water Purification System, Merck, India) water as a vehicle.

### Method of preparation

#### Preparation of NFRCS

Cryo-desiccation method was adopted for NFRCS development^11,12^. All the HFFC compositions were prepared (Table 1) using a mechanical stirrer. On a mechanical stirrer, 0.5% chitosan solution was prepared using 1% acetic acid in water by continuous stirring at 800 rpm. Accurately weighed quantity of supporting polymer mentioned in the Table 1 was added to chitosan solution and subjected to stirring until a clear polymeric solution was achieved. PVP K30 and PEG 400 were added to the obtained mixture as per the quantities mentioned in the Table 1 and kept for stirring until a clear, viscous polymeric solution was obtained. The obtained polymeric solution was bath sonicated for 60 min to remove the entrapped air bubbles from the polymeric mixture. As displayed in Supplementary Figure S1(b), Ct was evenly distributed in each well of 6 well’s plate (casting mold), and 5 mL of HFFC was added.

**Table 1:** Continuous phase composition of NFRCS.

S. No	Materials	Composition		
		Cm HFFC	Ch HFFC	Cp HFFC
1	Chitosan (%)	0.5	0.5	0.5
2	Methyl Cellulose (%)	0.5	–	–
3	HPMC 50 Cp (%)	–	0.5	–
4	PVA (%)	–	–	0.5
5	PVP K30 (%)	0.05	0.05	0.05
6	PEG 400 (%)	0.1	0.1	0.1
7	Cotton gauze (35 mm diameter) (mg)	200	200	200

The six-well plate was bath sonicated for 60 min to attain uniform wetting and distribution of HFFC in the network of Ct. Later, the six-well plate was frozen to −20 °C for 8–12 h. The frozen plate was cryo-desiccated for 48 h to achieve NFRCS of different compositions. The same procedure was used to produce different shapes and structures like tampons or cylindrical-shaped tampons, or any other shape for different applications.

### Preparation Chitosan Scaffold (Cs)

Accurately weighed chitosan (80 kDa) (3%) was dissolved in 1% acetic acid using a magnetic stirrer. To the obtained chitosan solution, 1% PEG 400 was added and left for stirring for 30 min. The obtained solution was transferred to a square or rectangular-shaped container and frozen at −80 °C for 12 h. The frozen sample was cryo-desiccated for 48 h to obtain porous Cs^13^.

#### In-vitro evaluation of NFRCS

### Chemical compatibility studies

The developed NFRCS were experimented with Fourier transform infrared (FTIR) spectroscopy (Shimadzu 8400 s FTIR, Tokyo, Japan) to confirm the chemical compatibility of chitosan with other polymers^14,15^. The FTIR spectrum of all test samples was obtained by executing 32 scans (spectral range width of 400 to 4000 cm^-1^).

#### Blood absorption rate

The blood absorption rate (BAR) for all the formulations was estimated using the method reported by Chen et al.^16^ with slight modifications. The developed NFRCS of all the compositions was dried in a vacuum oven at 105 °C for overnight to remove residual solvent. NFRCS 30 mg (initial weight of the sample—W_0_) and 30 mg of Ct (positive control) were placed in different petri dishes containing 3.8% sodium citrate premixed blood. At predetermined time intervals i.e., 5, 10, 20, 30, 40, and 60 s, NFRCS were removed, and their surface was cleaned to remove the unabsorbed blood by placing the samples on Ct for 30 s. The final weight of the NFRCS with absorbed blood was considered (W1) at each time point^16^. The percentage of BAR was calculated by using the following formula:

$${\text{BAR}} = \left[ {\left( {{\text{W}}_{1} - {\text{W}}_{0} } \right)/{\text{W}}_{0} } \right] \times 100\%$$

#### Blood clotting time

##### By using trisodium citrate

The blood clotting time (BCT) was determined by following the method reported by Wang et al.^17^*.* The time taken by the whole blood (3.8% sodium citrate premixed rat blood) to form a clot in the presence of NFRCS was considered BCT of the test sample. NFRCS (30 mg) of different compositions was placed in 10 mL of screw-cap vials and incubated at 37 °C. Blood (0.5 mL) was added to the vials, and 0.3 mL of 0.2 M CaCl_2_ was added to activate the blood coagulation. Finally, the vials were overturned (up to 180°) after every 15 s until a dense coagulum was formed. BCT of the sample was estimated based on the number of overturns of the vails^17,18^. Based on the BCT, the two best formulations were selected from NFRCS of Cm, Ch, and Cp for further characterization studies.

##### By using hemolysis

The BCT of Ch NFRCS and Cp NFRCS compositions was determined by implementing the method reported by Li et al*.*^19^. The Ch NFRCS, Cp NFRCS, and Cs (positive control) of 15 × 15 mm^2^ were placed in an individual petri dish (37 °C). The 3.8% sodium citrated blood was mixed with 0.2 M CaCl_2_ at a volume ratio of 10:1 to trigger coagulation. The 20 µL of 0.2 M CaCl_2_ mixed rat blood was added to the surface of samples and placed in an empty petri-plate. Blood dispensed in the empty petri-plate without Ct was considered as control. At regular time intervals of 0, 3, and 5 min, the coagulation was terminated by adding 10 mL of deionized (DI) water to a sample containing petri dishes without disturbing the blood clot. The uncoagulated red blood cells (RBC) undergo hemolysis in the presence of DI water and release hemoglobin. The hemoglobin was measured at different time points (HA (t)) by using a UV–Visible spectrophotometer at 540 nm (λ _max_ of hemoglobin). The absolute hemoglobin (AH (0)) absorbance of 20 µL of blood in 10 mL DI water at 0 min was considered a reference standard. The relative hemoglobin absorbance (RHA) of coagulated blood was calculated by HA (t)/ HA (0), using the same batch of blood.

### Tissue adhesion

Using the texture analyzer (Texture Pro CT V1.3 Build 15, Brookfield, USA), the adhesion property of NFRCS towards damaged tissue was determined. A cylindrically shaped mold with an open bottom was pressed against the inner side of the porcine skin (without any fatty layer). The samples (Ch NFRCS and Cp NFRCS) were applied via a cannula into the cylindrical-shaped mold to the porcine skin to form adhesion. After 3 min of incubation at room temperature (RT) (25 °C), the adhesion strength of NFRCS at a constant speed of 0.5 mm/sec was recorded^20^.

### Coagulation without blood loss

The key feature of the surgical sealant is to intensify blood coagulation with minimal blood loss. The coagulation without blood loss of the NFRCS was estimated using the previously reported method with few modifications^19^. A microcentrifuge tube (2 mL) (inner diameter of 10 mm) with orifices of 8 × 5 mm^2^ area was made on one side of the centrifuge tube (representing an open wound). NFRCS was used to close the orifice, and the transparent tape was used to seal the exterior margins. A 20 µL of 0.2 M CaCl_2_ was added to the microcentrifuge tube filled with 3.8% sodium citrate premixed blood. After 10 min, the microcentrifuge tubes were removed from the petri-plate, and the increased petri-plate weight owing to blood leaking from the NFRCS was determined (n = 3). Ch NFRCS and Cp NFRCS blood loss was compared to Cs.

### Wet integrity

As per the method reported by Mishra and Chaudhary^21^ with slight modifications, the wet integrity of NFRCS was determined. The NFRCS was placed in a 100 mL conical flask containing 50 mL water and swirled for 60 s without forming a vertex. The sample's physical integrity was visually inspected and prioritized as per acquisition^21^.

### Intactness of the surface coat

The binding strength of the HFFC with Ct was examined using a previously reported method with few modifications^22^. The intactness of the surface coat was estimated by exposing NFRCS to sonic waves (external stimuli) in the presence of milli Q water (Ct). The developed NFRCS of Ch NFRCS and Cp NFRCS were placed in a beaker containing water and bath sonicated for 1, 2, 3, 4, 5, 6, 7, 8, 9, 10, and 30 min, respectively. After drying, the percentage difference in the initial and final weight of the NFRCS was used to calculate the percentage of material loss (HFFC). The binding strength or loss of material from the surface was further supported by in vitro BCT. The binding efficiency of the HFFC towards Ct assures the blood coagulation and flexible coat on the surface on Ct^22^.

### Uniformity of NFRCS

The uniformity of developed NFRCS was determined based on the BCT of samples (30 mg) trimmed from randomly selected positions of total NFRCS. The previously mentioned BCT procedure was followed to determine the uniformity of the NFRCS. The degree of closeness among all five samples assures the uniformity of surface coat and deposition of HFFC in the Ct network.

### Nominal blood contact area

The nominal blood contact area (NBCA) was determined following the previously reported method with a few modifications^19^. 20 µL of blood was sandwiched between two surfaces of Ct, Ch NFRCS, Cp NFRCS, and Cs and allowed blood to coagulate. After 1 h, both the scaffold pieces were separated, and the clot area was measured manually. The average of three replicates was considered as the NBCA of NFRCS^19^.

### Dynamic vapor sorption

Dynamic vapor sorption (DVS) analysis was used to estimate the efficiency of NFRCS to absorb the moisture from the external environment or at the site of injury responsible for the initiation of coagulation. The DVS system estimates or records the uptake and loss of vapor from the sample gravimetrically using an ultra-sensitive balance with a mass resolution of ± 0.1 µg. Partial vapor pressure (relative humidity) was generated using an electronic mass flow controller around the sample by mixing saturated and dry carrier gases. As per the European Pharmacopeia guidelines, based on the percentage of moisture uptake by the samples, samples were categorized into 4 categories (0–0.012% w/w^−^ non-hygroscopic, 0.2–2% w/w slightly hygroscopic, 2–15% moderately hygroscopic, and > 15% very hygroscopic)^23^. The moisture-absorbing efficiency of NFRCS of Ch NFRCS and Cp NFRCS was determined by the TA TGA Q5000 SA DVS analyzer. During the process, the run time, relative humidity (RH), and real-time mass of the sample at 25 °C were obtained^24^. The moisture content was calculated by accurate mass analysis of the NFRCS by the following equation:

$${\text{Moisture content }}\left( {{\text{MC}}} \right) \, = {\text{ m}}_{{1}} - {\text{m}}_{{2}} /{\text{ m}}_{{2}} \times { 1}00$$

whereas MC is the moisture content of the NFRCS.m_1_ is the dry mass of the NFRCS.m_2_ is the real-time mass of the NFRCS at set RH.

### Brunauer–Emmett–Teller (BET) analysis

The total surface area was estimated using a nitrogen adsorption experiment with liquid nitrogen after emptying the samples at 25 °C for 10 h (< 7 × 10^–3^ Torr). The total surface area, pore volume, and pore size of NFRCS were determined by the Quantachrome, NOVA 1000e, Austria, with RS 232 software^25^.

### Erythrocyte agglutination test (EAgT)

The 5% erythrocytes (saline as diluent) were prepared from the whole blood. Later, the HFFC (0.25 mL) aliquots were transferred to a 96 well plate and a 5% erythrocyte mass (0.1 mL). The mixture was incubated at 37 °C for 40 min. The mixture of erythrocyte mass with serum was considered as a positive control, and saline with erythrocyte mass was considered as a negative control. Hem-agglutination was determined based on the Stayitsky scale. The proposed scale was as follows: +  +  +  + Compact granular agglutinate; +  +  + Smooth mat on the bottom of the well with folded edges; +  + Smooth mat on the bottom of well, edges ragged; + narrow red ring around the edge of smooth mat; - (negative) the discrete red button in the center of the bottom well^12^.

### Thrombogenicity

The hemocompatibility of NFRCS was studied by following the International Standard Organization method (ISO) (ISO10993-4, 1999)^26,27^. The gravimetric method reported by *Singh *et al*.* was implemented with few modifications to evaluate the thrombus formation in the presence or on the surface of NFRCS. The 500 mg of Cs, Ch NFRCS, and Cp NFRCS were incubated in phosphate buffer saline (PBS) at 37 °C for 24 h. After 24 h PBS was discarded, and the NFRCS was treated with 2 mL of 3.8% sodium citrated blood. To the NFRCS surface, 0.04 mL of 0.1 M CaCl_2_ was added to the incubated sample. After 45 min, the blood coagulation was terminated by adding 5 mL of distilled water. The coagulated blood on the surface of NFRCS was treated with 36–38% formaldehyde solution. The formaldehyde-fixed blood clots were dried and weighed. The percentage of thrombogenicity was estimated by calculating the beaker weight in the absence of blood and sample (negative control) and beaker weight with blood (positive control)^26^.

$${\text{Percentage}}\;{\text{thrombogenicity}} = \frac{{Mass{\text{ }}of{\text{ }}test{\text{ }}sample - Mass{\text{ }}of\left( { - ve} \right)control}}{{Mass{\text{ }}of\left( { + ve} \right)control - Mass{\text{ }}of\left( { - ve} \right)control}} \times 100$$

### Structural analysis

#### By using a light microscope

As a preliminary confirmation, samples were visualized under light microscopy to understand the ability of HFFC to surface coat, interconnect the Ct, and pore formation in the network of Ct. The thin section from NFRCS of Ch and Cp was trimmed using a surgical blade. The obtained section was placed on a glass slide, covered with a coverslip, and the edges were fixed with glue. The prepared slides were observed under a light microscope, and images were captured at different magnifications^28^.

#### By using a fluorescent microscope

The fluorescent microscopic technique was adopted to visualize the polymer deposition in the network of Ct, which is based on the method reported by Rice et al*.*^29^. The HFFC composition used for the formulation was mixed with a fluorescent dye (amaranth), and NFRCS (Ch & Cp) were prepared as per the method mentioned previously. From the obtained sample thin section of NFRCS was trimmed and placed on the glass slide, and covered with a coverslip. The prepared slide was visualized under a fluorescent microscope using a green filter (310–380 nm). The images were captured at 4× magnification to understand the Ct interconnection and excess polymer deposition in the network of Ct.

#### Surface morphology

The surface topography of NFRCS of Ch and Cp was determined in tapping mode using Atomic Force Microscope (AFM) with a super sharp TESP cantilever: 42 N/m, 320 kHz, 2–5 nm ROC, Bruker, Taiwan. The surface roughness was determined by root mean square (RMS) using software (scanning probe image processor). The various locations of NFRCS were visualized in 3D images to check the uniformity of the surface^30^. The standard deviation of the evaluation in the given area was defined as surface roughness. RMS equation was employed to quantify the surface roughness of the NFRCS^31^.

#### Field emission scanning electron microscope (FESEM) study and elemental analysis by Energy Dispersive Spectroscopy mapping

The FESEM based study was performed to understand the surface morphology of Ch NFRCS and Cp NFRCS (which showed better BCT than Cm NFRCS) by using FESEM, SU8000, HI-0876-0003, Hitachi, Tokyo. FESEM studies were performed as per the method reported by Zhao et al*.*^32^ with slight modifications. NFRCS of 20 to 30 mg of Ch NFRCS and Cp NFRCS were treated with 20 µL of 3.8% sodium citrate premixed rat blood. 20 µL, 0.2 M CaCl_2_ was added to blood-treated samples to initiate coagulation, and samples were incubated at RT for 10 min. Further, an excess amount of RBC was removed from the surface of the NFRCS by washing with saline.

Later samples were treated with 0.1% glutaraldehyde and subsequently dried at 37 °C in the hot air oven to remove the moisture. The dried samples were sputter-coated and subjected to analysis^32^. The other images captured during the analysis were clot formation on the surface of single cotton fiber, polymer deposition in between the Ct, the morphology of RBC (shape), clot intactness, and morphology of RBC in the presence of NFRCS. The area of untreated NFRCS and treated NFRCS of Ch and Cp incubated with blood were scanned for respective elemental ions (sodium, potassium, nitrogen, calcium, magnesium, zinc, copper, and selenium)^33^. The percentage of elemental ions was compared between treated and untreated samples to understand the accumulation of elemental ions in the clot formation and the uniformity of the blood clot.

#### Surface coat thickness by FESEM

The Cp HFFC surface coat thickness on the Ct surface was determined using FESEM. The transverse section of Cp NFRCS was trimmed from the scaffold and sputter coated. The obtained sputter-coated sample was observed under FESEM, and the thickness of the surface coat was measured^34–36^.

### X-ray micro-computed tomography (micro-CT)

The X-ray micro-CT provides high-resolution 3D non-destructive visualization and allows one to investigate the internal structural arrangement of NFRCS. The micro-CT uses an X-ray beam that passes through the sample to record the local linear attenuation coefficient for X-ray in the sample, which helps to yield the morphological information^37^. The internal arrangement of the Ct in Cp NFRCS and the blood-treated Cp NFRCS was studied by micro-CT to understand the imbibition efficiency and blood coagulation in the presence of NFRCS^37–39^. The 3D structure of blood treated and an untreated Cp NFRCS sample was reconstructed using micro-CT (V|tome|x S240, Phoenix, Germany). Multiple radiographs were generated from different angles (ideally covering 360°) to develop a 3D image of NFRCS by VG STUDIO-MAX version 2.2 software. The collected projections data were reconstructed into 3D volume images using suitable 3D simple ware ScanIP Academic software^37^.

Further, to understand the distribution of blood clots, 20 µL of premixed citrate blood was added to the NFRCS, and 20 µL 0.2 M CaCl_2_ was added to initiate blood coagulation. The prepared sample was left for coagulation. The surface of NFRCS was treated with 0.5% glutaraldehyde and dried in a hot air oven at 30–40 °C for 30 min. The blood clot formed on the NFRCS was scanned, and a 3D image of the blood clot was reconstructed and visualized.

### Antimicrobial assay

The antimicrobial assay was performed for the Cp NFRCS (which is best compared to Ch NFRCS) using a previously reported method with few modifications^40^. The antimicrobial activity of the Cp NFRCS and Cp HFFC was studied by using three different test micro-organisms [*Staphylococcus aureus (S. aureus)* (gram-positive bacteria), *Escherichia coli (E. coli)* (gram-negative bacteria), and *Candida albicans (C. albicans)*] which were grown on agar in petri dishes in an incubator. 50 mL of diluted levitation bacterial culture with a concentration of 10^5^–10^6^ cfu mL^−1^ was inoculated onto the agar culture medium uniformly. The culture media was poured into petri dishes and left for solidification. On the surface of agar spread plates, wells were made to fill the HFFC (3 wells for HFFC and 1 for a negative control). 200 µL of HFFC was filled in 3 wells, and 200 µL of pH 7.4 PBS was added to the 4^th^ well. On the other side of the petri dish, 12 mm discs of Cp NFRCS were placed on solidified agar and moistened with PBS (pH 7.4). The ciprofloxacin, ampicillin, and fluconazole drug discs were considered as reference standards for *S. aureus*, *E. coli*, and *C. albicans*. The zone of inhibition was measured manually, and digital images of the zone of inhibitions were captured.

### Thromboelastography (TEG)

After acquiring institution ethical approval, the study was conducted in education and research-based Kasturba Medical College, Manipal, Karnataka, India, a hospital in south India. The in vitro TEG experimental protocol was examined and approved by Institutional Ethical Committee, Kasturba Medical College, Manipal, Karnataka (IEC: 674/ 2020). The subjects from the voluntary blood donors (of age between 18 and 55 years) from the hospital blood bank were recruited. Furthermore, an informed consent form was taken from the volunteers for blood sample collection. Native TEG (N-TEG) was used to study the effect of Cp HFFC composition on sodium citrate premixed whole blood. N-TEG was well established for its role in point of care resuscitation, which poses challenges for clinicians due to delay in results time (conventional coagulation assay) which may cause clinical implications^41^. The N-TEG analysis was performed by using whole blood. Informed consent and detailed history were taken from all the participants. The participants with any hemostatic or thrombotic complications like pregnancy/ postpartum or liver disease were not involved in the study. The subjects on medication that influences the coagulation cascade were also excluded from the study. Basic laboratory tests (hemoglobin, prothrombin time, activated thromboplastin, and platelet count were performed for all the participants as per the standard procedure^42^. N-TEG determines the viscoelastic properties of  a blood clot, initial clot construction, pellet interaction, clot strengthening, and clot lysis. The N-TEG analysis gives the graphical and numerical output of the collective effect of several cellular elements and plasma^42^. The N-TEG analysis was performed for two different volumes of Cp HFFC (10 µL and 50 µL). The analysis was performed by adding 10 µL of Cp HFFC with 1 mL of whole citrated blood. A 1 mL of (Cp HFFC + citrated blood), 340 µL of blood from the mixture was added to 20 µL of 0.2 M CaCl_2_ containing TEG cup. Later, the TEG cup was loaded into a TEG® 5000, US to measure R, K, α-angle, MA, G, CI, TPI, EPL, LY 30% of the blood sample in the presence of Cp HFFC^41^.

### Pharmacodynamic studies

The in vivo studies protocol was reviewed and approved by the Institutional Animal Ethics Committee (IAEC), Kasturba Medical College, Manipal Academy of Higher Education, Manipal (IAEC/KMC/69/2020). All animal experiments were performed as per the Committee for the Purpose of Control and Supervision on Experiments on Animals (CPCSEA) guidelines. All in vivo studies of NFRCS (with size 2 × 2 cm^2^) were performed on female wistar rats (200 to 250 g). All the animals were acclimatized at 24–26 °C, and animals had free access to a standard chow diet and water ad libitum. All the animals were randomly separated into different groups, and each group consisted of three animals. All the studies were performed in accordance with the Animal Research: Reporting of in vivo Experiments^43^. Before the study, the animals were anesthetized with an intraperitoneal (IP) injection of 20 to 50 mg ketamine (per 1 kg body) and 2–10 mg xylazine (per 1 kg body weight) cocktail. After the study, hemorrhagic volume was calculated by estimating the difference in the initial and final weight of the sample; the average value obtained from three trials was considered as the hemorrhagic volume of the sample^44^.

### Rat tail amputation model (external injury model)

The rat tail amputation model was implemented to understand the potential of NFRCS in regulating bleeding in external injuries, combats, or road accidents (external injury model). A 50% of the tail was amputated using the surgical blade and left for 15 s in the air to ensure normal bleeding. Further, a test sample was placed on the rat tail by applying pressure (Ct, Cs, Ch NFRCS, and Cp NFRCS). The hemorrhagic volume and BCT of the test samples were recorded (n = 3)^17,45^.

### Superficial femoral artery model

The pressure-controlling efficiency of the NFRCS in combats was studied using a superficial femoral artery model. The femoral artery was exposed and punctured with a 24G cannula needle and allowed to bleed for 15 s. After observing uncontrollable bleeding, the test sample was positioned at the punctured site by applying pressure. After immediate application of the test sample, the clotting time was recorded, and hemostatic efficiency was observed for the next 5 min. The same procedure was repeated with Cs and Ct^46^.

### Rat liver injury model

Dowling et al*. *^47^ proposed a liver injury model to estimate the hemostatic potential of the hemostatic material in intraoperative bleeding conditions. The BCT of Ct (negative control), Cs scaffold (positive control), Ch NFRCS, and Cp NFRCS samples was recorded. By performing midline laparotomy, the super-hepatic vena cava of the rat was exposed. Later, the left distal lobe portion was excised using scissors. An incision was made on the liver with a surgical blade and allowed to bleed for a few seconds. Accurately weighed test samples of Ch NFRCS and Cp NFRCS were placed on the injury surface without any positive pressure, and BCT was recorded. Subsequently, the control group (Ct) was studied by applying pressure, followed by Cs without disturbing the injury for 30 s^47^.

### Wound healing assay by excision model

In vivo wound healing assay was performed to estimate the wound healing property of the developed polymer-based NFRCS using the excision wound model. The excision wound model was opted and performed as per the method previously reported with few modifications^19,32,48^. All the animals were anesthetized, as stated earlier. A circular deep cut injury was made on the dorsal flank skin using a biopsy punch (12 mm). The prepared wound area was dressed in Cs (positive control), Ct (to understand cotton adhesion interferes with the healing), Ch NFRCS, and Cp NFRCS (test groups), and a negative control without any treatment. On every alternative day of the study, the wound area of all the rats was measured. The wound area was captured using a digital camera and dressed with a new dressing. The percentage of wound closure was measured by using the following formula:

$${\text{Wound}}\;{\text{closure}}\left( \% \right) = \left( {{\text{area}}\;{\text{of}}\;{\text{the}}\;{\text{wound}}\;{\text{on}}\;{\text{the}}\;{\text{nth}}\;{\text{day}}/{\text{initial}}\;{\text{area}}\;{\text{of}}\;{\text{the}}\;{\text{wound}}\;{\text{on}}\;0{\text{th}}\;{\text{day}}} \right) \times 100$$

### Hematoxylin and Eosin (H & E) staining and Masson's trichrome staining

Based on the percentage of wound closure on the 12th day of the study, the rat skin of the best group was excised ((Cp NFRCS) and the control group) and studied by H& E staining and Masson's trichrome staining. The staining procedures implemented were followed as per the previously reported methods^49,50^. In brief, following 10% formalin fixation, samples were dehydrated using graded alcohol series. A thin section (5 μm thickness) of excised tissue was obtained using a microtome. The thin serial section of control and Cp NFRCS was treated using hematoxylin and eosin to study the histopathological changes. Masson's Trichrome staining was employed to identify the collagen fibers formation^49^. The results obtained were investigated in a blinded manner by a pathologist.

### Stability studies

The stability of the Cp NFRCS sample was studied at room temperature (25 °C ± 2 °C/ 60% RH ± 5%) for 12 months^51^. Cp NFRCS was inspected visually (color change and microbial growth on the surface) and tested for fold endurance and BCT as per the above method elucidated in the materials and methods.

### Scale-up

The scalability and reproducibility of Cp NFRCS was studied by preparing 15 × 15 cm^2^ size Cp NFRCS. Further, from various portions of the Cp NFRCS, 30 mg of samples were trimmed (n = 5), and the BCT of the test samples was estimated, as stated earlier under methodology.

### Different applications

We attempted to develop various shapes and structures using Cp NFRCS composition for various biomedical applications. The shape or structure includes cone-shaped tampons to control nasal bleeding, dental surgeries, and cylindrical-shaped tampons to control vaginal bleeding.

### Statistics

All the data sets were expressed as mean ± SD and analyzed by ANOVA followed by Bonferroni multiple comparison tests (∗ p < 0.05) using Prism 5.03 (GraphPad, San Diego, CA, USA).

### Approval for human experiments

All procedures performed in research involving human participants were performed by following the institute standards and national research committee and with the 1964 Helsinki Declaration and its later amendments or comparable ethical standards. All the participants were informed about the characteristics of the study and its voluntary nature. The participant data was maintained confidential after collection. The in vitro TEG experimental protocol was examined and approved by Institutional Ethical Committee, Kasturba Medical College, Manipal, Karnataka (IEC: 674/ 2020). For blood sample collection, the volunteer has signed an informed consent form.

### Approval for animal experiments

All procedures performed in research involving animals were performed by following the Kasturba Medical College, Manipal Academy of Higher Education, Manipal (IAEC/KMC/69/2020). All animal experiments designed were as per the Committee for the Purpose of Control and Supervision on Experiments on Animals (CPCSEA) guidelines. All the authors complied with the ARRIVE guidelines.

## Results

### Chemical compatibility studies

**Figure 2:**
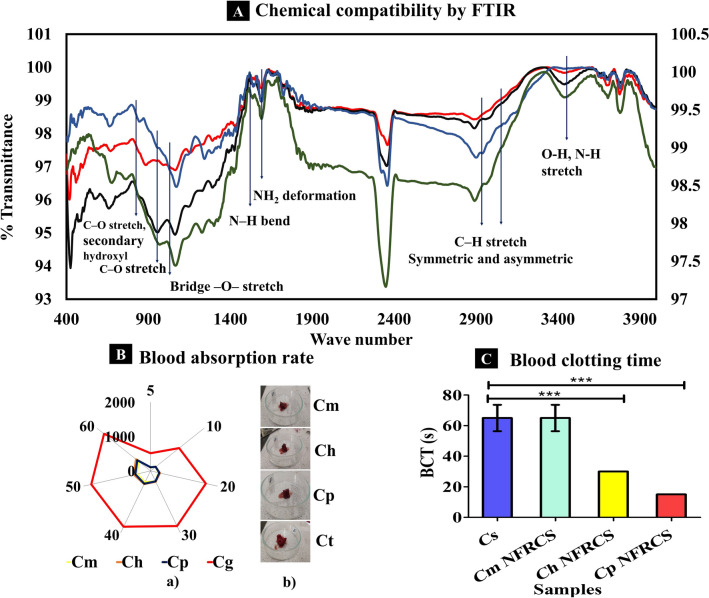
In vitro characterization features of Cm NFRCS, Ch NFRCS, Cp NFRCS, and Cs. (**A**) Represents the combined FTIR spectrum of chitosan and Cm NFRCS, Ch NFRCS, and Cp NFRCS formulations in compression. (**B**) a) The whole blood absorption rate of NFRCS of Cm, Ch, Cp, and Cg, (n = 3); The Ct sample showed high BAR as the cotton gauge has high imbibing efficiency; b) Pictorial representation of blood-absorbed samples after blood absorption. **C** The graphical representation of BCT of test samples (Cp NFRCS has the best BCT (15 s, n = 3)). Data in C, D, E, and G were shown as mean ± SD, and the error bars represent SD, ***p < 0.0001.

The FTIR spectrum of all the NFRCS was analyzed and compared with the chitosan spectrum shown in Fig. 2A. The characteristic spectral peaks of chitosan (reported) were found at 3437 cm^-1^ (O–H stretch and N–H stretch, overlapped), 2945 and 2897 cm^-1^ (C-H stretch), 1660 cm^-1^ (NH_2_—deformation), 1589 cm^-1^ (N–H bend), 1157 cm^-1^ (bridge -O- stretch), 1067 cm^-1^ (C–O stretch, secondary hydroxyl group), 993 cm^-1^ (C-O stretch, primary -OH)^52–54^. Supplementary Table S1 represents the FTIR absorption spectral values of chitosan (reported), pure chitosan, NFRCS of Cm, Ch, and Cp. The FTIR spectrum of all the NFRCS (Cm, Ch, and Cp) has shown characteristic absorption bands like pure chitosan without any significant changes (Fig. 2A). The FTIR results confirm no chemical or physical interaction between polymers used to develop NFRCS, which depicts the inert nature of the polymers used.

### Blood absorption rate

The NFRCS, with excellent blood absorption rate support, controls the massive unmanageable bleeding from the injury. Figure 2B shows the blood absorption rate of Cm NFRCS, Ch NFRCS, Cp NFRCS, and Ct (positive control), and 2B (b) represents the images captured during the study. All the NFRCS and Ct engorged up to 60 s; however, the BAR of Ch NFRCS (539.38 ± 14.14 mg) was more significant than Cm NFRCS (444.42 ± 91.58 mg) and Cp NFRCS (493.92 ± 83.69). The obtained results of Ct (1731.93 ± 10.20 mg) exhibited more BAR than other test samples. A significant difference was observed between Cm NFRCS, Ch NFRCS, and Cp NFRCS with ***p < 0.0001%.

### Blood clotting time

#### By using trisodium citrate premixed blood

The hydrophilicity of the hemostatic material or polymer is an essential factor in designing surgical sealants or hemostatic scaffolds^16^. The in vitro BCT helps to understand the effect of HFFC to promote coagulation at the site of application. The BCT of Cs scaffold, Cm NFRCS, Ch NFRCS, and Cp NFRCS was 65 ± 8.66, 65 ± 8.66, 30 ± 0.00, and 15 ± 0.00 s, respectively. Among all the samples, Cp NFRCS showed less BCT. The comparative results were depicted graphically in Fig. 2C. Supplementary Figure S3 represents BCT and images captured during the study. Further, the hemolysis and BCT were also determined based on the hemolysis method^19^. A significant difference in blood clotting time was found with *p* < 0.0001% between Cs, Ch NFRCS, and Cp NFRCS, whereas no significant difference was observed between Cm and Cs; Cp NFRCS and Ch NFRCS.

### Based on hemolysis

**Figure 3:**
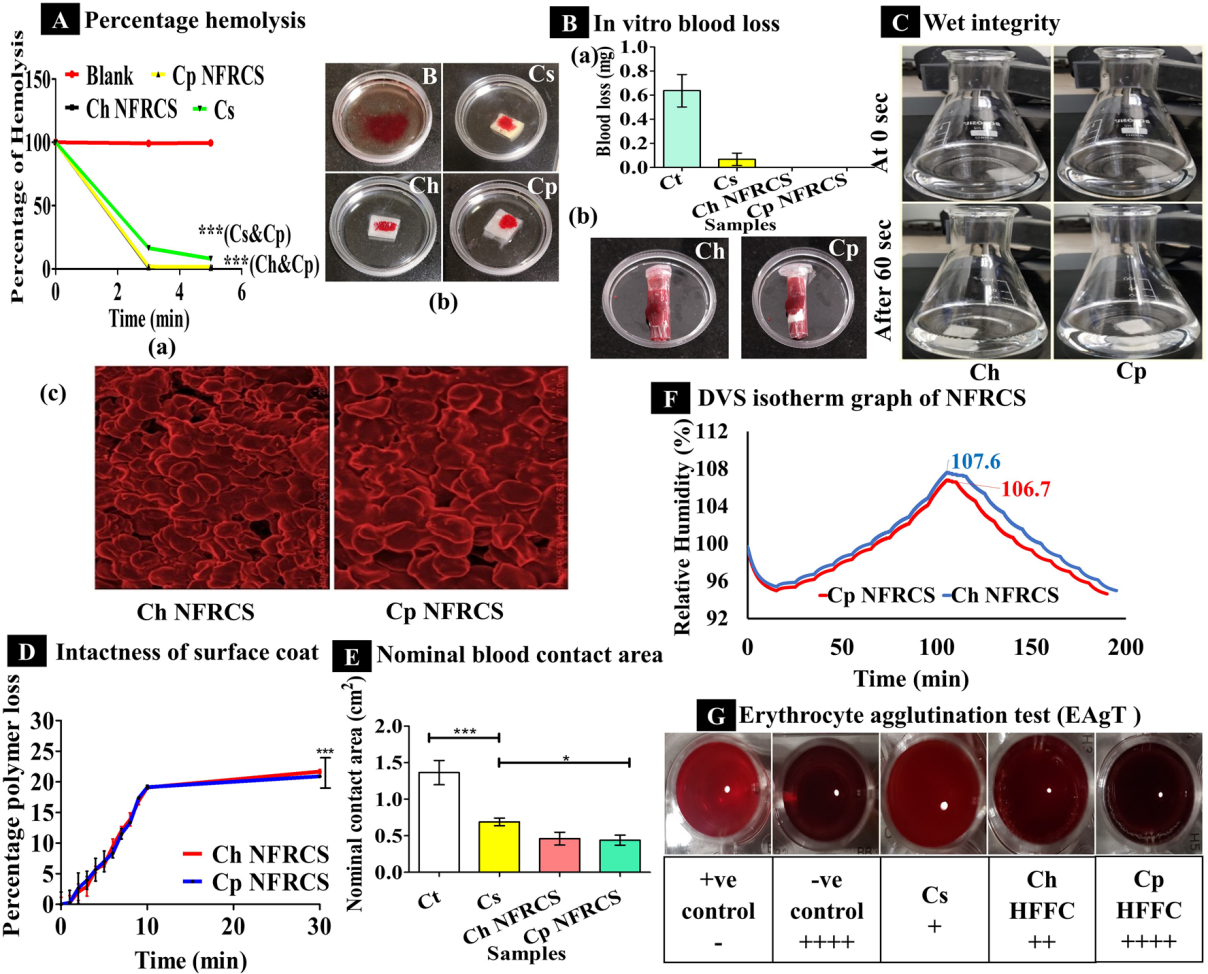
The above image represents the in vitro evaluation of Ch NFRCS, Cp NFRCS, and Cs. (**A**) a) Graphical representation of percentage hemolysis of RBC in the presence of Ch NFRCS, Cp NFRCS, and Cs, (n = 3); b). Images captured during the hemolysis assay; c). Represent the FESEM images captured from the surface of blood-treated Cp NFRCS and Ch NFRCS. (**B**) a) Amount of blood loss before blood coagulation in the presence of Ct, Ch NFRCS, Cp NFRCS, and Cs samples (n = 3), no blood loss was observed for Ch NFRCS and Cp NFRCS; b) The digital images captured during the study of Ch NFRCS and Cp NFRCS samples in vitro blood coagulation before blood loss. (**C**) The above digital images represent the wet integrity of Ch NFRCS and Cp NFRCS at 0 s and 60 s (n = 3). (**D**) Graphical representation of intactness of the surface coat when exposed to external stimuli (sonic waves) (n = 3). (**E**) Graphical representation of NBCA of test samples (n = 3). (**F**) The above image represents the DVS isotherm of Ch NFRCs and Cp NFRCS samples (hygroscopic). (**G**) The image represents the Erythrocytes agglomeration in the presence of Cs, Ct, Ch HFFC, and Cp HFFC samples (Cp HFFC showed the best EAgT compared to other samples). Data in A, B, D, and E images is represented as mean ± SD, n = 3, with ***p < 0.0001.

BCT of the NFRCS was determined and compared with Cs. As per the ISO 10993–4 guidelines, based on the percentage of hemolysis, biomaterials can be classified into three different categories, hemolytic (> 5%), slightly hemolytic (2 to 5%), and non-hemolytic (< 2%)^55^. The Ch NFRCS and Cp NFRCS were non-hemolytic (percentage of hemolysis < 2%), and Cs was hemolytic (percentage hemolysis is > 5%). The RHA (t) at 3 min and 5 min were 1.41 ± 0.352%, & 0.99 ± 0.246%, 1.81 ± 0.017% & 1.70 ± 0.007%, 17.33 ± 0.779%, & 8.29 ± 0.177% of Ch NFRCS, Cp NFRCS and Cs, respectively (Fig. 3Aa). Figure 3A(b) represents RBC lysis in the presence of blank and test samples. Figure 3A(c) represents the observations captured by the FESEM, which shows the intact structure of RBC. The smaller the RHA (t), the quicker the clot formation in the presence of biomaterial, the lesser the hemolysis of RBC. The obtained results of test samples (Cs and Cp NFRCS; Ch NFRCS and Cp NFRCS) for hemolysis showed a significant difference with ***p < 0.0001%.

### Adhesion properties

Blood loss and strong clot adhesion are serious problems associated with the hydrophilic hemostatic materials or scaffolds^19^. The adhesion property of the NFRCS was determined. Both Ch NFRCS and Cp NFRCS showed minimal adhesion with a peak load of 43 g and 75 g. Usually, cotton gauge adheres to wet surface or damaged tissue after blood coagulation or slow drying of the wound surface, interferes with the wound healing process as the wound gets distributed while changing the dressing. Minimal tissue adhesion of NFRCS assures the uniform surface coat of the Ct. On the 2^nd^, 4^th^, or 6^th^ day of wound healing assay, NFRCS did not exhibited any tissue adhesion, which further confirmed the tissue adhesion property of NFRCS. Supplementary Figure S2 Represented the tissue adhesion system-generated plots.

### Coagulation without blood loss

Coagulation without blood loss of Ch NFRCS and Cp NFRCS was determined. The blood loss from Ch NFRCS and Cp NFRCS was compared with Ct (negative control) and Cs (positive control). The results showed that blood coagulation was instantaneous without any blood loss in the case of Ch NFRCS and Cp NFRCS. Ct and Cs showed 0.64 g and 0.06 g of blood loss (Fig. 3Ba). The nanoporous (< 5 nm) and FRC structure of Ch NFRCS and Cp NFRCS did not allow blood to flow out through the orifice of the microcentrifuge tube. Figure 3B(b) represents the digital images captured during the study of Ch NFRSC and Cp NFRCS.

### Wet integrity

The results of the wet integrity test indicate the physical strength of the structure when it meets or interacts with the blood or wound exudates^21^. The change in the physical structure like softening or formation of gel or break down of polymeric structure at the site of application and the removal of dressing from the site of injury also implicates the ease of patient compliance. Figure 3C represents the images captured during the study that indicates Ch NFRCS and Cp NFRCS were intact or maintained the structure, ensuring the ease of removal and can withstand the back pressure excited by the blood.

### Intactness of the surface coat

The intactness of the surface coat was determined, which assures the intactness of the surface of the NFRCS during transportation, packing, storage, and application. As the HFFC (Ch and Cp) were composed of water-soluble polymers and excess polymer deposited on the top surface of the Ct, 21.69 ± 0.30% (Ch NFRCS) and 20.91 ± 0.15% (Cp NFRCS) of HFFC loss was observed after 30 min of exposure to sonic waves. Minimal loss of HFFC was recorded after 30 min due to the compact structure of the NFRCS. Figure 3D represents the intactness of the surface coat plot (percentage HFFC loss from NFRCS on the Y-axis and time on the X-axis was plotted). A significant difference between Ch and Cp NFRCS was observed with ***p < 0.0001%.

### Uniformity of NFRCS

The uniformity of NFRCS of Ch NFRCS and Cp NFRCS was determined. The average BCT was 65 ± 6.70 and 15 ± 0.00 s of Ch NFRCS and Cp NFRCS. The five different samples trimmed from different portions of 15 × 15 cm^2^ showed similar BCT, confirming the uniform distribution of HFFC.

### Nominal blood contact area

The NBCA of NFRCS was determined; the Ct absorbed the blood quickly compared to Ch NFRCS and Cp NFRCS. Cp NFRCS (11.00 ± 1.73%) showed less NBCA than Ch NFRCS (11.5 ± 2.17) and Cs (17.25 ± 1.29). Figure 3E represents the NBCA of the Ch NFRCS, Cp NFRCS, and Ct. Blood coagulation in the presence of hemostatic material could be one factor that influences the NBCA of the material or NFRCS (NBCA is directly proportional to the BCT of the NFRCS). A significant difference was observed between Cs and Cp NFRCS with *p < 0.05%.

### Dynamic vapor sorption

The moisture uptake ability of the NFRCS was estimated by DVS analysis. Figure 3F represents the moisture content uptake by Ch NFRCS and Cp NFRCS with the varying RH levels over the time profile during the isotherm run. Concerning the European Pharmacopeia guidelines, based on the percentage moisture gain by the NFRCS, it can be concluded that Ch NFRCS and Cp NFRCS were hygroscopic. The percentage of moisture uptake by Ch NFRCS and Cp NFRCS was 7.6% and 6.8% to the initial weight. Both the samples have similar moisture uptake ability (< 1%) due to HFFC composition (both Cp NFRCS and Ch NFRCS contain 0.05% PVA K30). PVA consists of hydrophilic hydroxyl groups which interact with water; similarly, in the case of HPMC, it consists of hydrophilic hydroxypropyl substitutions and hydrophobic methoxyl groups, which exhibit low hygroscopicity^56^.

### Brunauer–Emmett–Teller (BET) analysis

Supplementary Table S3 represents the BET results of Cp NFRCS and Ch NFRCS results. The total surface area of Ch NFRCS and Cp NFRCS was found to be 20.479 m^2^/g and 11.295 m^2^/g, respectively. The average pore diameter of Cp NFRCS and Ch NFRCS was < 3 nm.

### Erythrocyte agglutination test

The EAgT of Ch HFFC, Cp HFFC, and 3% Cs solution was determined. Erythrocytes displayed complete or compact granular agglutination (+ +  + +) without any supernatant in the presence of Cp HFFC and negative control. The Cs results were in accordance with the previously reported results^12^. Ch HFFC displayed a smooth mat on the bottom of the well with edges ragged (+ +) with little supernatant. Figure 3G shows EAgT images of all the test samples.

### Thrombogenicity

The non-thrombogenic nature of the scaffold is one of the critical factors of biomaterials or hemostatic materials. The percentage of thrombogenicity of the Cs, Ch NFRCS, and Cp NFRCS were 4.63 ± 0.23%, 2.33 ± 0.11%, and 2.65 ± 0.39%, respectively (Supplementary Figure S5). Cs, Ch NFRCS, and Cp NFRCS exhibited a significant difference with p < 0.0001%. The Ch NFRCS and Cp NFRCS showed less percentage of thrombogenicity than the Cs scaffold, which confirms the non-thrombogenic nature of the developed NFRCS. Other factors include hydrophilicity, chemical composition, surface topology, and morphology that influence the surgical sealants' hemolytic potency^57,58^.

### Structural analysis

#### By using a light microscope

**Figure 4:**
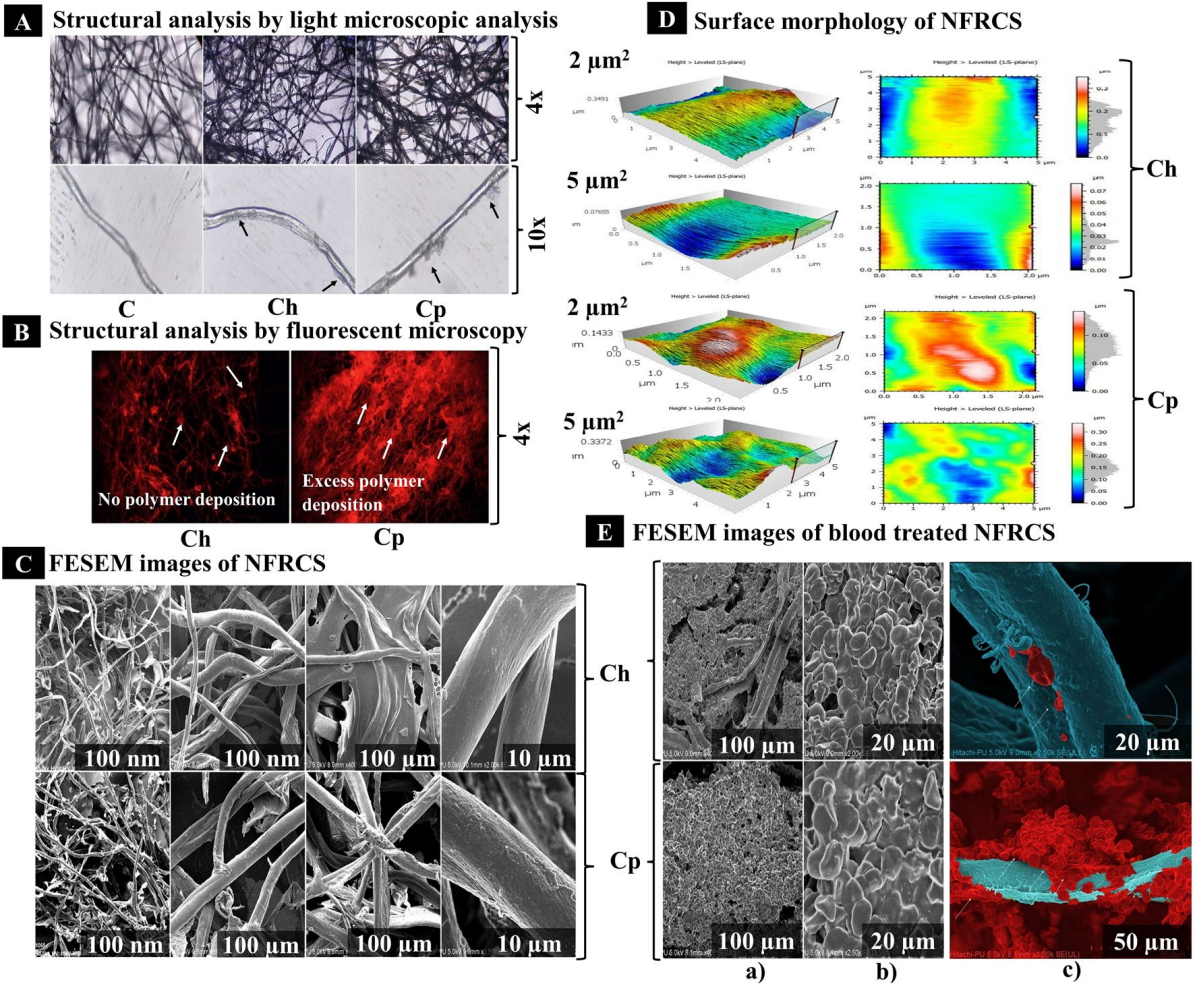
The structural analysis of Ch NFRCS and Cp NFRCS by variuos techniques. (**A**) Represents **t**he HFFC deposition on the surface of the Ct and excess polymer deposition in the network of fibers interconnection (micrograph captured at 4 × and 10x). (**B**) The microscopic images (4x) represent the HFFC disposition in the network of Ct by using a fluorescent microscope. (**C**) The FESEM images show the Ct fiber's interconnection, film formation among the Ct fibers, and uniform surface coating on the surface of Ct fibers. (**D**) represents the surface morphology (surface roughness) of Ch NFRCS and Cp NFRCS at 2 µm and 5 µm height using AFM. 3D images were reconstructed using MountainsLab® Premium 8.2.9526 (https://www.digitalsurf.com/support/software-updates/), and at 2 µm height, Ch showed better surface smoothness than Cp NFRCS. (**E**) The FESEM images show the blood clot formation on the surface of NFRCS, intactness of the RBC clot, clot formation on the surface of Ct at submicron level, and formation of polyhedrocytes confirm the intactness of the clot in the presence of Cp NFRCS.

The 4× and 10× microscopic images of NFRCS of Ch and Cp showed an HFFC deposition (thin layer) on the surface of Ct and polymer deposition in the network of the Ct (Fig. 4A). The Cp NFRCS showed more polymer deposition on the surface of Ct than Ch NFRCS.

#### By using a fluorescent microscope

The images captured at 4× revealed that Cp NFRCS showed better interconnection of Ct and excess HFFC deposition in the network of Ct (the intense fluorescence produced by the HFFC between the Ct). Figure 4B represents the fluorescent micrograph of Ch NFRCS that displays no excess HFFC deposition on the Ct surface and in between the Ct network. The Cp NFRCS fluorescent micrograph displayed excess polymer deposition between the Ct network and surface (Fig. 4B).

#### Surface morphology

Using AFM, the surface morphology of the NFRCS was studied. The topology of NFRCS was scanned at 2 and 5 µm^2^. Figure 4C and D represent the surface morphology and structural framework of Ch NFRCS and Cp NFRCS, respectively. The white color region represents the protrusions on the NFRCS surface, and the other colors like red, green, and blue represent the depressions of the NFRCS. The 3D images generated using MountainsLab® Premium 8.2.9526 provided decent depth to understand the surface of the NFRCS. Overall, the surface topology obtained from the AFM suggests that Ch NFRCS (RMS value of 2 µm is 0.011 and 5 µm is 0.059) exhibited more surface smoothness compared to Cp NFRCS (RMS value of 2 µm is 0.024 and 5 µm is 0.044).

#### Field Emission Scanning Electron Microscope (FESEM)

The morphology of Ch and Cp NFRCS and blood-treated Ch NFRCS and Cp NFRCS samples were studied using FESEM. The images obtained from FESEM at different magnifications confirm the uniform coating of HFFC on the surface of the Ct, and HFFC was able to interconnect the randomly arranged Ct. Figure 4C shows the nano (n) to micron (µ) size film projections on the surface of Ch NFRCS and Cp NFRCS. Figure 4B shows the excess polymer deposition between the Ct network reflected in the formation of NFRCS. The random Ct arrangement facilitated achieving the uniform NFRCS with nanopore size (< 3 nm).

Further, the blood-treated Ch NFRCS and Cp NFRCS showed intact clot formation on the surface of NFRCS (Fig. 4Ea). The clot formation or more RBC adhesion on the surface of Ct was more in the case of Cp NFRCS compared to Ch NFRCS surface (Fig. 4Ea and b). The modified FESEM images of Ch NFRCS and Cp NFRCS showed the uniform nano thickness film hemostatic surface coat, and clot formation was observed in the submicron level as depicted in Fig. 4E(c). The RBC present on the Ch NFRCS remained intact, confirming the HFFC compatibility with RBC. On the other hand, the RBC present on the surface of Cp NFRCS displayed a change in the morphology (polyhedral shape) which were named polyhedrocytes (Fig. 4Eb). The formation of polyhedrocytes on the surface of Cp NFRCS confirms the clot's intactness. Further, the polyhedrocytes improve the mechanical strength of the clot. The intact clot has the potential to alter the permeability of the thrombolytic enzymes to dissolve the clot^59^.

#### Elemental analysis by Energy Dispersive Spectroscopy mapping

**Figure 5:**
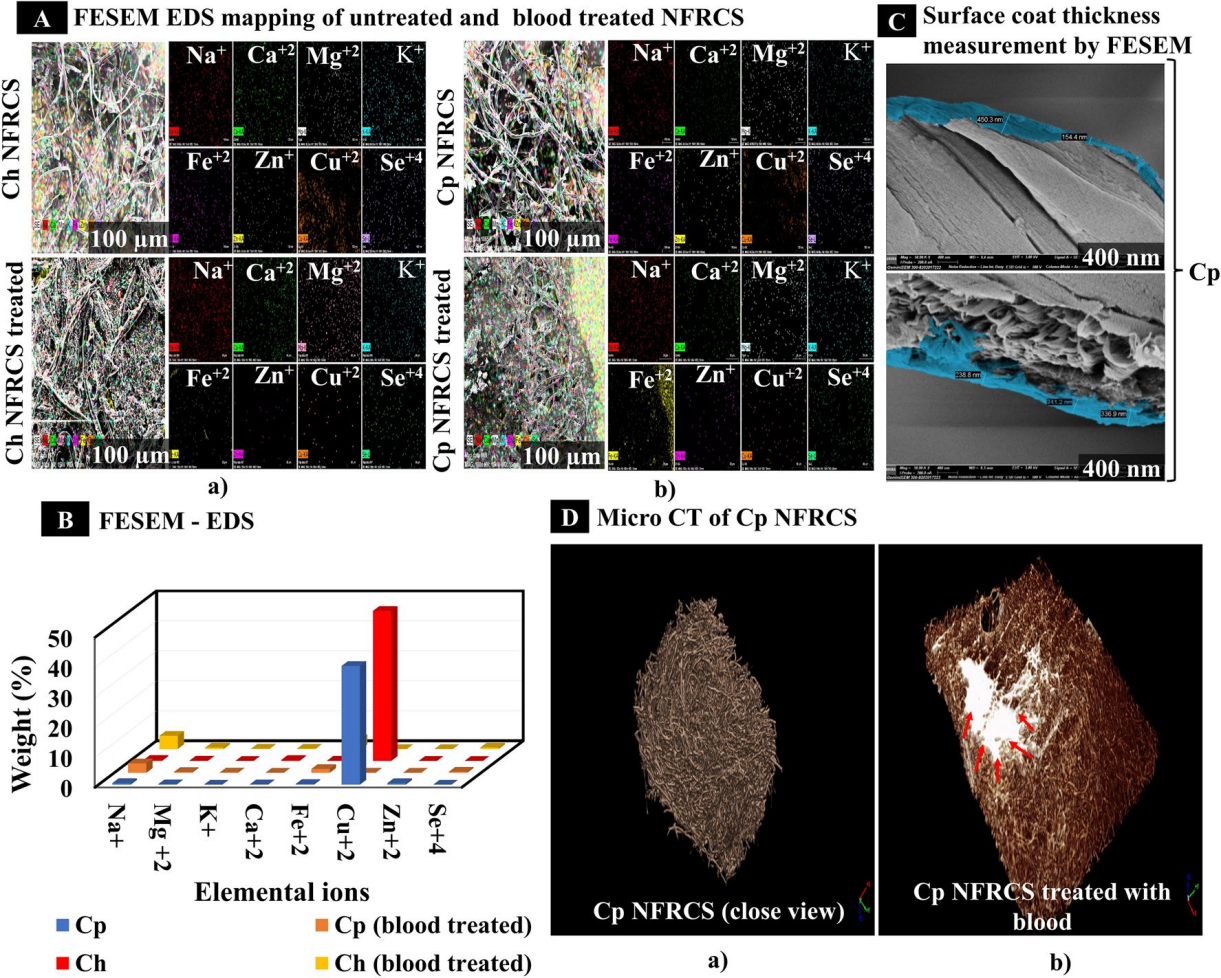
FESEM, EDS mapping, and micro- CT of NFRCS samples. (**A**) a). FESEM-EDS mapping of Ch NFRCS and Ch NFRCS treated with blood; b). FESEM-EDS mapping of Cp NFRCS and Cp NFRCS treated with blood. (**B**) Graphical representation of elemental ions mapped in blood treated and untreated NFRCS of Ch and Cp, (**C**) Thickness of surface coat on the surface of Ct by using FESEM (the HFFC was able to form nano thickness film ranges from 150 to 500 nm). (**D**) a). Represents the 3D simple ware ScanIP Academic software (https://www.synopsys.com/simpleware/software.html) modified micro-CT image of a close view of NFRCS; b). The image represents the modified micro-CT image of Cp NFRCS after blood treatment.

The elemental ions Na^+^, Mg^2+^, K^+^, Ca^2+^, Fe^2+^, Cu^2+^, Zn^+^, and Se^4+^, were mapped on the surface of NFRCS of Ch NFRCS, Cp NFRCS, and Ch NFRCS, Cp NFRCS treated with blood. The elemental mapping results showed an increase in elemental ions accumulation in the blood clot, from which we can understand clot formation was excellent and uniform. Excess Na^+^ ions concentration was due to premixed sodium citrate blood used for the study. The FESEM mapped images showed in Fig. 5A(a) and (b) uniform distribution of elemental ions in blood-treated Ch NFRCS and Cp NFRCS, respectively. Along with Ca^2+^, previously published reports concluded that Mg^2+^, Na^+^ play an essential role in blood coagulation^60,61^. Mg^2+^ ions accelerate the activation of factor X by factor IXa in the presence of factor VIIIa, phospholipid, and Ca^2+^ ions and increase the catalytic activity^61^. The Na^+^ ions help thrombin significantly activate fibrinogen and protease-activated receptors (PAR1) and trigger the initial build-up factor Va, VIIa, and XIa, which generate thrombin. Sezer et al*. *^62^ reported K^+^ ions cross-linked oxidized regenerated cellulose showed better hemostatic activity when compared to Ct, which reflects K^+^ ions cross-linking improved the blood clotting ability^62^. Zn^+^ ions play a vital role in platelet aggregation, hemostasis, anticoagulation, and fibrinolysis.

Consequently, zinc acts as a switch to regulate coagulation and inhibits the process by activating compensatory inhibitory pathways^63^. Metal ions in the NFRCS (untreated sample) were due to Ct used to develop NFRCS. A previously reported study states that Ca^2+^, Na^+^, Mg^2+^, Cu^2+^, Zn^+^, K^+^ were dominant in Ct^64^. In the previous report published, authors speculated that Cu^2+^ is essential for maintaining the heterodimeric structure at physiologic factor VIII concentrations^65^. The copper ions increase the activity of factor VIIIa^66^. The copper ions bridge the A1 and A3 domains of Factor VIII to enhance the affinity by ∼100 folds, maintaining the hydrodynamic structure of factor VIII concentration^65^. The Se^4+^ is a vital micro immunonutrient that holds the individual's metabolic activity with chemical bonding. Glutathione peroxidase is one of the significant selenoproteins which supports the regulation of excessive production of free radicals at the site of injury or inflammation^67^. The additional elemental ion found in mapping is Fe^2+^; ceruloplasmin converts Fe^2+^ ferrous to the Fe^3+^ (ferric ion), which helps to bound by transferrin^68^. Fe^3+^ ions result in the formation of insoluble fibrin clot formation^69^. Except for Ca^2+^, Zn^2+^, and Se^4+^ ions, an increase in the elemental ion's concentration was observed in blood-treated samples (Figure 5Aa and b). Figure 5B represents the number of elemental ions present in untreated and blood-treated NFRCS of Ch and Cp. An increase in ions concentration might be due to the accumulation of micro elemental ions in the blood involved in the blood coagulation cascade. The increase in the elemental ion concentration confirms the uniformity and natural clot formation in the presence of NFRCS.

#### Surface coat thickness

Based on the above studies (BCT, clot formation on the surface of Cp surface coated Ct fiber (Fig. 4E), we conclude that Cp NFRCS was the best HFFC composition. Further, the thickness of the surface coat of the HFFC was estimated by FESEM. The thickness of the film coat of Cp HFFC on the surface of Ct ranges from 150 to 500 nm. Figure 5C represents the modified FESEM images of the nano thickness film surface coated on the surface of Cp NFRCS.

### X-ray micro-CT scanning

The obtained results confirmed no void spaces or uneven Ct distribution in the processed micro-CT image of Cp NFRCS (Fig. 5Da). We could not analyze the pore size using micro-CT because the Cp NFRCS pore size was < 3 nm. Figure 5Db represents the image of blood-treated Cp NFRCS. The in vitro BAR showed that the entire NFRCS could absorb blood, and the entire NFRCS was red in color. The modified micro-CT image of Cp NFRCS (Fig. 5Db) showed white color (blood absorbed), in which blood coagulation was initiated instantaneously by Cp HFFC due to which blood did not spread to the entire area of NFRCS.

### Antimicrobial assay

**Figure 6:**
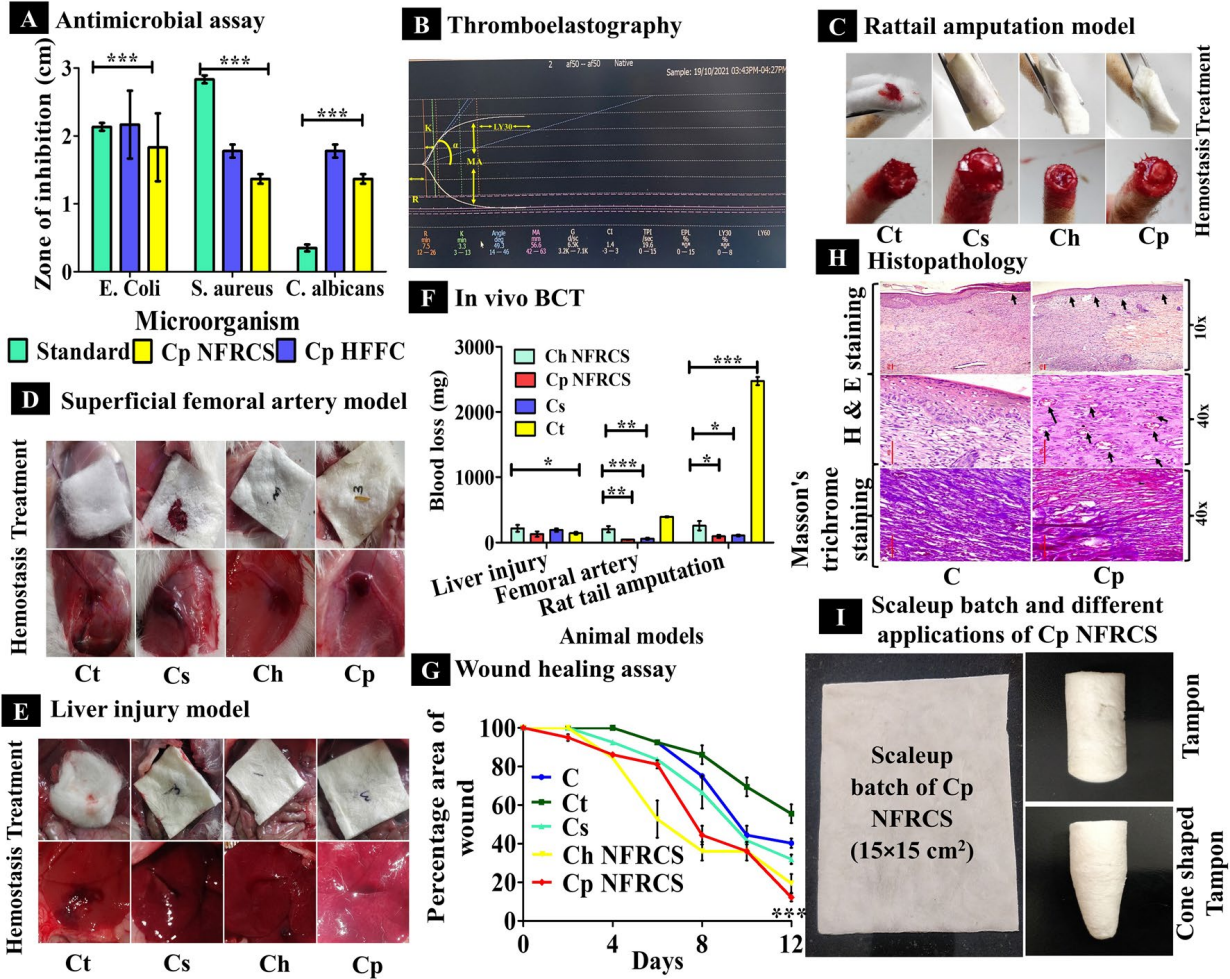
Various in vitro and in vivo characterization studies of NFRCS. (**A**) The graph represents the Cp HFFC and Cp NFRCS antimicrobial activity against three different micro-organisms. (**B**) Represents the N-TEG system-generated graph of Cp HFFC, which shows a reduction in R-value, indicating the quick initiation of blood coagulation in the presence of 10 µL Cp HFFC. (**C, D**, **E**) Represent the images captured during treatment and hemostasis after placing dressing material of various samples during in vivo BCT of tail amputation, superficial femoral artery, and liver injury model, respectively. (**F**) The graph represents the BCT of NFRCS in all animal models. (**G**) The graph represents the comparative wound healing profile of C, Ct, Cs, Ch NFRCS, and Cp NFRCS on the excision wound model; Cp NFRCS showed the best wound closure percentage (12.22 ± 0.25%) compared to other samples. (**H**) Represents the micrographs of C (control) and Cp NFRCS (showed better healing progress) stained by H&E and Masson's trichrome staining. Epidermis and blood vessels formation was seen in Cp NFRCS treated group, and mature collagen formation was observed in Cp NFRCS by Masson trichrome staining. (**I**) The images represent the scale-up batch of Cp NFRCS (15 × 15 cm^2^), tampon, and cone shape tampon developed for different biomedical applications. Data in A, F, and G is represented as mean ± SD, n = 3 with *p < 0.05.

Microbial infections are one of the significant concerns which may cause severe complications. Both Cp HFFC and Cp NFRCS were active against all the test micro-organisms. Figure 6A represents the antimicrobial activity of Cp HFFC and Cp NFRCS against *S. aureus, E. coli,* and *C. albicans*. The digital images captured during the study of Cp HFFC and Cp NFRCS exhibited a clear zone of inhibition (Supplementary Figure S6). The Cp NFRCS has more zone of inhibition against *S. aureus* and *C. albicans* than *E. coli,* whereas Cp HFFC exhibited more zone of inhibition than Cp NFRCS. The Cp HFFC could diffuse more into the nutrient agar media than Cp NFRCS. The zone of inhibition of Cp NFRCS was 15.53% less than the Cp HFFC for *E. coil* and 23.16% less for *S. aureus* and *C. albicans*. As the NFRCS was intact and HFFC entrapped in the Ct network, it could not diffuse from the dispersed phase. Further, the zone of inhibition change was due to structural differences in the cell wall of micro-organisms. The gram-negative bacterium (*E. coli*) cell wall consists of a bilayer with an outer lipopolysaccharide layer, making it difficult for HFFC to inactivate or show its activity^70^. Cp HFFC displayed a significant difference with ***p < 0.0001% compared Cp NFRCS.

### Thromboelastographic

The viscoelasticity property of the human blood with Cp HFFC (10 µL and 50 µL) was determined. N-TEG curve and clotting parameters, including reaction time (R), α angle, and maximum amplitude of Cp HFFC (10 µL and 50 µL) with whole blood, were compared. N-TEG curve displayed no difference in the R-value of Cp HFFC at two different concentrations ((< 0.1 min), 10 µL (7.4 min), 50 µL (7.5 min)), and the control sample exhibited more R-value (9.1 min) than Cp HFFC. The R-value of control was less than the reference range (12–26 min); this might be due to the clotting factor’s activation after the subject's blood withdrawal. The Cp HFFC could significantly intensify the coagulation rate, despite the minimal amount of polymeric material. In the presence of Cp HFFC, whole blood showed hypercoagulation (based on R-value 10 µL (7.4 min), 50 µL (7.5 min)), which is less than the reference range (12–26 min) irrespective of Cp HFFC concentration. The increase in Cp HFFC volume exhibited an increase in α- the angle. The firmness of the clot (G) was more for 10 µL Cp HFFC (7.2 d/sc) compared to 50 µL Cp HFFC (6.5 d/ sc) and control (6.4 d/ sc). The MA value of 10 µL of Cp HFFC (58.9 mm) was more than the 50 µL and control (56.1 mm), indicating more platelets aggregation during blood coagulation. The increase in Cp HFFC volume (50 µL) resulted in a decrease in MA value due to platelet consumption in the clot formation. Figure 6B represents the system-generated N-TEG graph of 10 µL Cp HFFC. Supplementary Figure S7 represents the system-generated graph of control and 50 µL HFFC. Supplementary Table S4. shows all the N-TEG values of Cp HFFC (10 µL), Cp HFFC (50 µL), whole blood (control).

### Pharmacodynamic studies

The Ch and Cp NFRCS exhibited preferable in vitro BCT. The pharmacodynamic activity of the developed NFRCS was studied in various animal models to understand and estimate the efficacy of the composition in intraoperative and external injuries. The BCT and blood loss in all the animal models (rat liver puncher model, rat tail amputation model**,** and superficial femoral artery model) were estimated and presented in Supplementary Figure S8.

### Rat-tail amputation model (external injury model)

The BCT of the test samples was estimated in the rat tail amputation model. The BCT of Ct, Ch NFRCS, Cp NFRCS, and Cs was 367 ± 18.08 s, 194.66 ± 14.57 s, 124.33 ± 8.71 s, and 175 ± 3.21 s, respectively. The BCT of Cp NFRCS was less than Ch NFRCS, Cs, and Ct. in vivo blood loss of Cp NFRCS was less than Ch NFRCS, Cs, and Ct (95.3 ± 23 mg, 258 ± 72.10 mg, 108 ± 13.52 mg, and 2473 ± 62.20 mg, respectively). Cs and Cp NFRCS exhibited a significant difference in BCT with *p < 0.05%. Blood loss was more in the case of cotton compared to Cp NFRCS, Ch NFRCS, and Cs. Figure 6C displays the in vivo images captured during the rat tail amputation study.

### Superficial femoral artery model

The BCT of the test samples was estimated in the rat femoral artery model. As represented in Fig. 6D femoral artery was exposed and was punctured with a 24 G needle. The Ch NFRCS, Cp NFRCS, Cs, and Ct exhibited BCT of 104.33 ± 12.01 s, 40 ± 5.56 s, 50 ± 7.54 s, and 124.33 ± 24.00 s, respectively. BCT was significantly less in Cp NFRCS than in other test samples (Cp NFRCS and Ch NFRCS; Cs and Cp NFRCS ***p < 0.0001%). Cp NFRCS showed a significant difference in blood loss (45.30 ± 3.05 mg), compared to Ch NFRCS, Cs, and Ct (205 ± 48.60 mg, 59.70 ± 14.84 mg, and 394 ± 9.00 mg respectively) with *p < 0.05%. Cp NFRCS had the lowest BCT and blood loss of all the test samples, indicating high Cp NFRCS coagulation efficiency.

### Rat liver injury model

The BCT of the test samples was determined in the rat liver injury model. Figure 6E and 6F represent the images of the liver injury model captured during the study and BCT of the test samples. The BCT of Ct, Ch NFRCS, Cp NFRCS, and Cs was 124 ± 18.08 s, 138.33 ± 14.57 s, 59 ± 8.71 s, and 126.33 ± 3.21 s, respectively. The in vivo blood loss of Ct, Ch NFRCS, Cp NFRCS, and Cs test samples was 130.70 ± 10.08 mg, 218 ± 52.84 mg, 131.00 ± 41.04 mg, and 191.30 ± 14.57 mg, respectively. Cp NFRCS has less BCT with minimal blood loss among all the compositions. Cs showed better BCT than Ch NFRCS with less blood loss. During the animal study, no pressure was applied to restrict blood flow from the liver injury, demonstrating that the Cp NFRCS might be employed effectively for non-compressible lesions (internal injuries or intraoperative bleedings). Cs, Ch NFRCS, and Cp NFRCS displayed a significant difference with ***p < 0.0001%. Cp NFRCS showed superior hemostatic efficiency in all three animal models with less hemorrhagic blood loss among all the test samples. The Cp HFFC composition-initiated coagulation as the HFFC consists of PVA and PVP K 30, which were highly hydrophilic compared to HPMC and chitosan. In contrast, the Cp HFFC could absorb blood and accumulate many erythrocytes and platelets on the injury surface to form a physical barrier (Fig. 4Eb)^45^. Figure 6F represents the comparative BCT graph of all the animal model samples.

### Wound healing assay by excision model

NFRCS was used to treat full-thickness wounds (12 mm) shown in (Supplementary Figure S9). Usually, Ct interferes with the wound healing by sticking to an open wound surface and may cause pain while removing or changing dressing^71^. The effect of Ct was studied and compared with the effect of NFRCS. Gross observation of full-thickness wounds in rats showed a significant reduction in the percentage of wound area of Cp NFRCS, Ch NFRCS, and Cs (12.22 ± 0.25%, 19.44 ± 0.57%, and 31.94 ± 0.28%) treated groups compared to C and Ct groups. On the second day of the experiment, Ch NFRCS, Cs, and Cp NFRCS scaffold-treated groups displayed dry wounds, which confirms that the Cp NFRCS, Ch NFRCS, Cs scaffold could absorb moisture, maintain the microenvironment by exchange of gases, and also regulated inflammation^50^. On the 12th day of the study, Cp NFRCS treated group exhibited the maximum wound closure area (12.22 ± 0.25%) than other test sample treated groups.

On the surface of Ch NFRCS and Cp NFRCS treated wounds, no cotton fibers adhered (no tissue adhesion) that confirmed the uniform surface coating of the Ct by the HFFC. In agreement with the results of gross observation, Fig. 6G displays the wound healing assay graph. The HFFC composed of 0.5% PVA and chitosan possesses a faster wound healing rate than C, Ct, Cs, and Ch NFRCS groups. The results reveal that the high wound closure rate of the Cp NFRCS treated wounds could contribute to better inflammation and antimicrobial control at the initial stage of the wound healing process^50^. Further, the Cp NFRCS treated skin sample was excised, and histopathology of skin samples was studied to understand the effect of HFFC composition in the formation of epithelium, granulation, and mature collagen formation. A significant difference was observed between the test samples (Cs and Ch NFRCS *p < 0.01%; Cs and Cp NFRCS *p* < 0.0001%).

### Hematoxylin and Eosin (H& E) staining and Masson's trichrome staining

The control (C) and Cp NFRCS treated group skin samples were excised and stored in 10% formalin for 48 h and studied by Hematoxylin and Eosin (H & E) and Masson's trichrome staining. In the Cp NFRCS treated group, the newly formed epidermis was thick and healthy without any interference between host tissue and newly developed tissue, indicating that Cp HFFC favored tissue regeneration and extracellular matrix development. The epithelium organization in Cp NFRCS treated group closely resembles the natural skin due to stable immobilization and sustained release of the PVA and chitosan at the application site. The C group exhibited incomplete or unorganized epithelial tissue (Fig. 6H) within the interference between the regenerated and host tissue, indicating poor wound healing. The 40× image captured of the Cp NFRCS treated group showed granulation in tissue with newly formed blood vessels (Fig. 6H) and proliferation of blood vessels (blue color) (Fig. 6H). The 40× image of the Cp NFRCS group showed the organization of macrophages in granulation tissue and epithelium formation above the granulation tissue (Fig. 6H). The C group showed an improper scab area above the granulation tissue (Fig. 6H). The 40× image of the C group showed proliferating fibroblasts cells (red), blood vessels, and acute inflammatory cells. After Masson's trichrome staining, the Cp NFRCS treated group exhibited improved mature collagen formation with fiber deposition and alignment.

### Stability studies

The stability of Cp NFRCS at room temperature was determined. There was no change in the Cp NFRCS compactness or mechanical strength. The folding endurance was > 1000 times. The BCT of the stability sample of Cp NFRCS was like the fresh sample (15 ± 0.00 s). There was no microbial growth or color change, or release of fibers from the Cp NFRCS, which confirms the stability of the Cp NFRCS. Supplementary Figure S10 and Table S6 represent the Cp NFRCS reference sample image and stability data.

### Scale-up

#### Uniformity of pilot formulation by using BCT

The BCT of developed pilot-scale Cp NFRCS (15 × 15 cm^2^ size NFRCS) of five different portions was determined (Fig. 6I). The average BCT of five different portions of the scale-up batch was 15 s ± 0.00 (Supplementary Table S5).

### Applications

The Cp HFFC composition was able to mold as per the structure of the casting mold and able to form the structure as per the design. The obtained tampon or cone-shaped structure or nasal tampon confirms the ability of HFFC, and some compositions can also be used for developing different structures for various biomedical applications. Figure 6I shows the modified forms of NFRCS (tampon and cone-shaped tampon) for different biomedical applications. Cp NFRCS can be modified into wrist bands by surface coating the adhesive layer, which helps to save lives in the application of deep-cut wrist injuries.

## Discussion

The study aimed to develop NFRCS using Ct as a reinforcing material and engineered to attain topological nanoporous bioactive scaffolds and nano thickness film-coated Ct with minimum polymer utilization for different biomedical applications (Fig. 6I). The nanostructure engineered surface can help to achieve fast hemostasis at the site of application^19^. NFRCS achieved enhanced and hastened coagulation by disintegrating tiny film structures present on the surface (Fig. 4C) of a dispersed phase in a pool of blood at the application site.

The Cp HFFC compositions resulted in fast blood coagulation and intact clot formation without blood loss (Fig. 3Bb). NFRCS showed minimal in vitro and in vivo tissue adhesion, which prevents secondary bleeding while undressing from the healing or injured tissue, which meets the ease of patient compliance. The minimal tissue adhesion prevents severe problems due to the strong adhesion of the clot plaguing the application of hydrophilic dressing materials. The NFRCS acts as a drug delivery templet and a hemostatic base substratum. Other classes of active molecules can be loaded into a nano thickness film, which improves transdermal drug delivery systems and widens their applications. Indeed, it can be used for bullet injuries by increasing the thickness of the NFRCS to customize it for different sites at the lower limb, neck, and other sensitive locations of the subject's body. The surface coat on the Ct can withstand even when exposed to mechanical or external stress. The Cp NFRCS exhibited promising antimicrobial activity, which significantly prevents microbial attacks.

Due to this, Cp NFRCS maintains a sterile zone at the application site and reduces the load of pathogenic microbiota and its spread, one of the significant concerns of intraoperative, combat, or road accidents. Due to its biocompatibility and proven in vitro assays qualitatively, the NFRCS was compatible and safe in internal and external injuries applications. The nanostructuring approach (pore size < 3 nm) helps to protect the tissue from direct exposure to the external environment or dust particles with proper gases exchange and breathability. NFRCS can be modified into roll form, which is simple and easy to carry. Cp NFRCS exhibits high efficacy and better wound healing ability when compared to Cs (pure chitosan) by maintaining the optimum moisture content and gases exchange during wound healing, which makes NFRCS attribute a multifunctional surgical sealant. The studies reveal and demonstrate that nanostructured surface helps in fast clotting with minimal blood loss-free and exhibit less tissue adhesion. Considering all the above qualitative attributes elucidated, the nanoporous and nano thickness film-forming, multifunctional material produced in this research offers the potential to advance the state of the art of hemostatic or wound dressing. NFRCS can be used for various Biomedical, Bioengineering, and molecular, cellular therapy applications. Cellulose fibers are an alternative for fiber reinforcement for implants and internal use. With this accomplishment, the study and material design strategy or approach developed by following adopted methods help to analyze and increase the scope of developing more efficient biomedical products, increasing the number of discoveries in the field of Biomedical & clinical applications to save humankind.

## Conclusion

The current research engineered NFRCS with nanoporous structure and nano thickness film surface-coated to Ct with its complete in vitro and in vivo characterization to determine the hemostatic and wound healing activities. The studies conclude that the NFRCS engineered by Ct fiber reinforcing material exhibited good topology with physical and mechanical strength with excellent hemostatic and wound healing properties. The whole concept is a masterpiece that can design and develop NFRCS prototypes with different topological features. The proposed design can be implemented in the fields of Biomedical Sciences and Bio-Engineering for various applications.

## Supplementary Information

Supplementary Information.

## Data Availability

All the datasets supporting the findings of this research are available from the corresponding author upon reasonable request.
