# Supplementary material for: Nanoporous and nano thickness film-forming bioactive composition for biomedical applications
Source: Sci Rep. 2022 May 17;12:8198. doi: 10.1038/s41598-022-12280-8 (PMC9114407; doi:10.1038/s41598-022-12280-8)
Supplement: Supplementary file 1 — Supplementary Information. [file 41598_2022_12280_MOESM1_ESM.docx]

**Supplementary information**

**Nanoporous and Nano Thickness Film-Forming Bioactive Composition for Biomedical Applications**

Naga Thirumalesh Chevala^1^, Lalit Kumar^1*^, Vimal Veetilvalappil^2^, [Aranjani Jesil Mathew](https://manipal.pure.elsevier.com/en/persons/jesil-mathew-a)^2^, Bemma Paonam^4^, Ganesh Mohan^4^, Shamee Shastry^4^, Krishnan Balasubramanian^5^, C Mallikarjuna Rao^3^

^1^Department of Pharmaceutics, ^2^Department of Biotechnology, ^3^Department of Pharmacology, ^4^Department of Immunohematology and Blood transfusion, ^5^Department of Mechanical Engineering,

^1,2,3^ Manipal College of Pharmaceutical Sciences, ^4^Kasturba Medical College, Manipal Academy of Higher Education, Manipal, Karnataka, India.

^5^Indian Institute of Technology, Madras, India

Email: [lalit.kumar@manipal.edu](mailto:lalit.kumar@manipal.edu)

**Supplementary Tables & Figures**


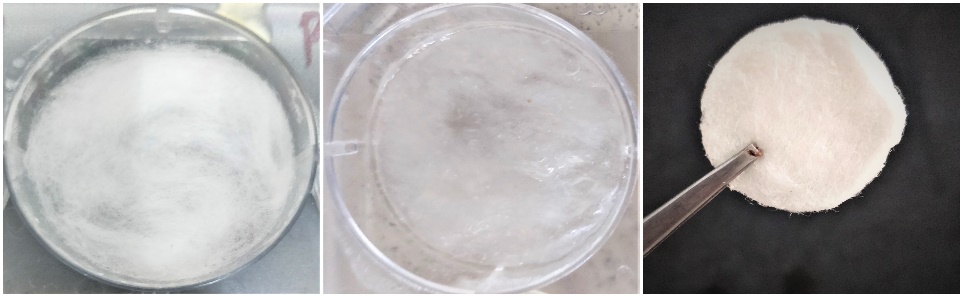


**a)**

**b)**

**c)**

**Supplementary Figure S1** a). The above image shows the arrangement of a dispersed phase (Ct) in a six-well plate, b). Represents the Ct incubated with HFFC and c). The image represents the NFRCS after lyophilization. The random arrangement of Ct improves the mechanical strength and results in the formation of nanoporous scaffold ^1^. It helps in tissue regeneration and maintains the microenvironment at the application site ^2^. It also offers a large surface area with different topography, which helps to adjust the surface chemistry ^1^.

**Supplementary Table S1.** Comparison of FTIR absorption spectral values of all samples

| Functional groups | Wavenumber (cm^-1^) | | | | |
| --- | --- | --- | --- | --- | --- |
|  | **Chitosan reported** | **Chitosan** | **Cm NFRCS** | **Ch NFRCS** | **Cp**  **NFRCS** |
| O-H stretch and N-H stretching | 3435 | 3437 | 3444 | 3435 | 3435 |
| C-H stretch | 2922, 2871 | 2945, 2897 | 2962, 2891 | 2953, 2889 | 2937, 2899 |
| NH_2_ deformation | 1656 | 1660 | 1653 | 1654 | 1660 |
| N-H bend | 1603 | 1600 | 1591 | 1587 | 1591 |
| Bridge -O- stretching | 1160 | 1157 | 1140 | 1145 | 1139 |
| C-O stretching, secondary hydroxyl group | 1085 | 1064 | 1064 | 1062 | 1070 |
| C-O stretching,  primary -OH | 1030 | 1028 | 1028 | 1031 | 1030 |

**Supplementary Table S2.** *In vitro* characterizations of NFRCS, All the NFRCS were homogenous and uniform. The Cp NFRCS sample has more weight than Cm NFRCS, and Ch NFRCS might be due to excess polymer deposition in the network of the dispersed phases. The surface pH of all the NFRCS was 5.38 to 5.49 (slightly acidic). In the presence of NFRCS, a change in morphology of RBC or hemolysis of RBC (Fig. 2B(c)) was not observed.

| Formulations | Visual appearance | Surface  uniformity | Thickness  (mm) | Weight  variation (mg) | Surface pH |
| --- | --- | --- | --- | --- | --- |
| Cm NFRCS | Homogenous | Uniform | 2.51 ± 0.02 | 158.04 ± 1.98 | 5.38 ± 0.01 |
| Ch NFRCS | Homogenous | Uniform | 2.53 ± 0.01 | 154.97 ± 1.69 | 5.45 ± 0.03 |
| Cp NFRCS | Homogenous | Uniform | 2.53 ± 0.02 | 167.41 ± 1.68 | 5.49 ± 0.06 |

**Supplementary Figure S2.** System generated plot of tissue adhesion of Ch and Cp NFRCS. The minimal tissue adhesion prevents the secondary bleeding while removing the dressing material from the application site after bleed coagulation and preventing possible infections at the application ^3^.

**C**

**Cs**

**Cm NFRCS**

**NFRCS**

**Ch NFRCS**

**Cp NFRCS**


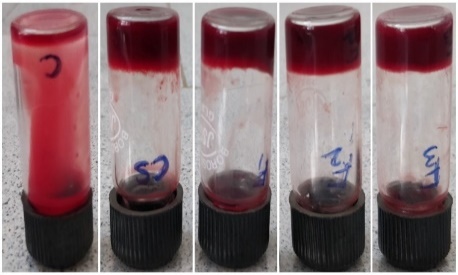

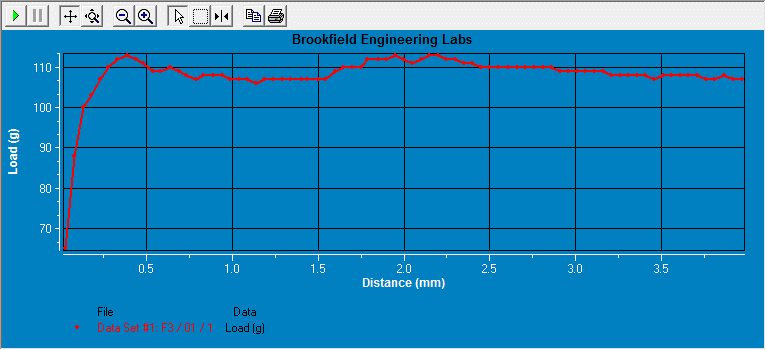

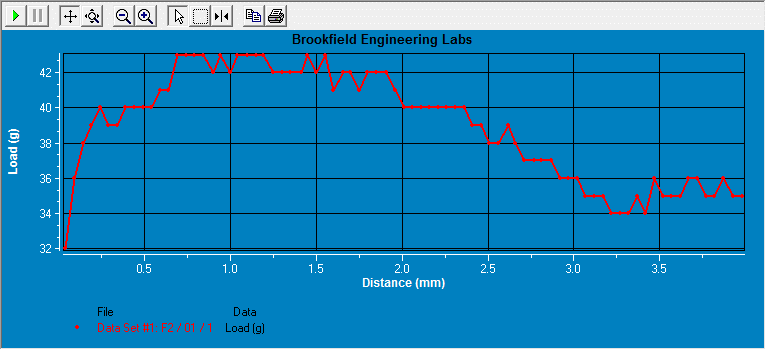


**Ch NFRCS**

**Cp NFRCS**

**Supplementary Figure S3.** represents the in vitro blood coagulation images in 5 mL culture of C, Cs, Cm NFRCS, Ch NFRCS, and Cp NFRCS samples (n=3). BCT of Cs, Cm NFRCS, Ch NFRCS, and Cp NFRCS was intact with dense mass clot formation. No clot formation was observed in C (control, sodium citrate mixed blood).

**Cp NFRC**

**Ch NFRCS**

**Cs**


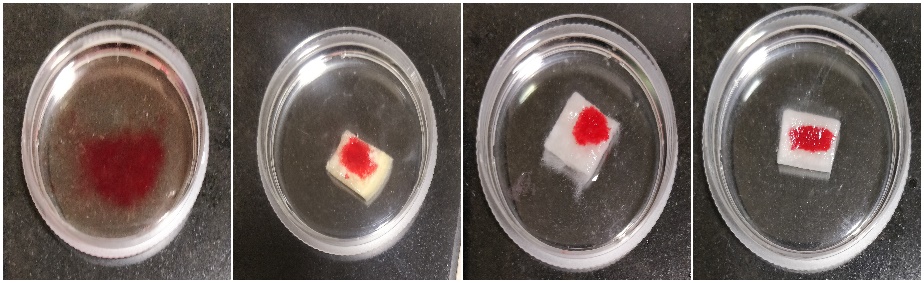


**Blank**

**Supplementary Figure S4.** Represents the images of hemolysis of RBC in the presence of different samples (blank, Cs, Ch, and Cp scaffolds), n=3. The Cs, Ch HFFC, and Cp HFFC compositions were compatible with blood, as minimal hemolysis of RBC was recorded (Fig. 3A(a)).

**Supplementary Table S3.** BET analysis of the surface area, pore-volume, pore diameter of Ch NFRCS, and Cp NFRCS. BET analysis confirms the nanoporous scaffold formation

| **Formulation** | **Surface area (m^2^/g)** | **Pore volume (cc/g)** | **Pore diameter (nm)** |
| --- | --- | --- | --- |
| Ch NFRCS | 20.479 | 0.056 | 2.195 |
| Cp NFRCS | 11.295 | 0.028 | 2.562 |

**Supplementary Figure S5.** The percentage thrombogenicity of Cs, Ch NFRCS, and Cp NFRCS samples. Thrombogenicity is one of the critical factors of biomaterial, which helps to understand the thrombus formation in the presence of biomaterial. The percentage thrombogenicity of Ch NFRCS (2.33 ± 0.11%) and Cp NFRCS (2.65 ± 0.39%) (no significant difference was observed) and the percentage is less than Cs. Data represented as mean ± SD, n=3, ***p < 0.0001.

**Supplementary Figure S6.** Antimicrobial assay of Cp HFFC and Ch NFRCS against *E. coli, S. aureus,* and *C. albicans*, n=3, p < 0.05, respectively. The report published by *Luigina Cellini et al.* states that S. aureus (37%), followed by *Pseudomonas aeruginosa* (17%), Proteus mirabilis (10%), E.coli (6%), and Corynebacterium species (5%) are the most common wound infecting microorganisms ^4^. On the other hand, fungal microorganisms are also prominent in infecting wounds, resulting in chronic wounds. The most commonly identified fungi are *Candida species* and *Cladosporium species* ^5^. Based on the previous reports, we shortlisted E. coli (gram -ve), S. aureus (gram +ve), and C. albicans (which cause fungal, which is uncommon but causes severe infection) to understand the antimicrobial activity of Cp NFRCS. A clear zone of inhibitions was observed in the presence of Cp HFFC and Cp NFRCS (Supplementary Fig. S6). The Cp HFFC composition and Cp NFRCS were active against all three organisms, confirming the continuous phase's antimicrobial nature.


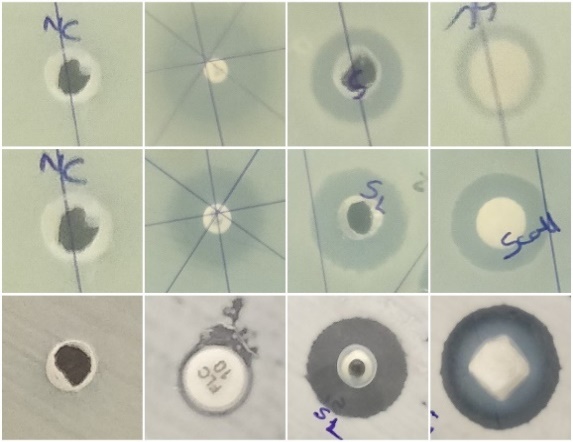


Pbs

Standard

(Drug disk)

Cp

(HFFC)

Cp

NFRCS

E. coli

S. aureus

C. albicans

| Parameters | Reference range | Control | Cp HFFC 10 µL | Cp HFFC 50 µL |
| --- | --- | --- | --- | --- |
| **R (min)** | 12 - 26 | 9.1 | 7.4 | 7.5 |
| **K (min)** | 3 - 13 | 3.2 | 3.2 | 3.3 |
| **α-angle** | 14 - 46 | 48.4 | 48.5 | 49.3 |
| **MA (mm)** | 42 - 63 | 56.1 | 58.9 | 56.6 |
| **G (d/sc) (K)** | 3.2 – 7.1 | 6.4 | 7.2 | 6.5 |
| **CI** | -3 - 3 | 0.9 | 1.8 | 1.4 |
| **TPI/ sec** | 0 - 15 | 19.6 | 22.0 | 19.6 |

**Supplementary Table S4.** N**-**TEG analysis


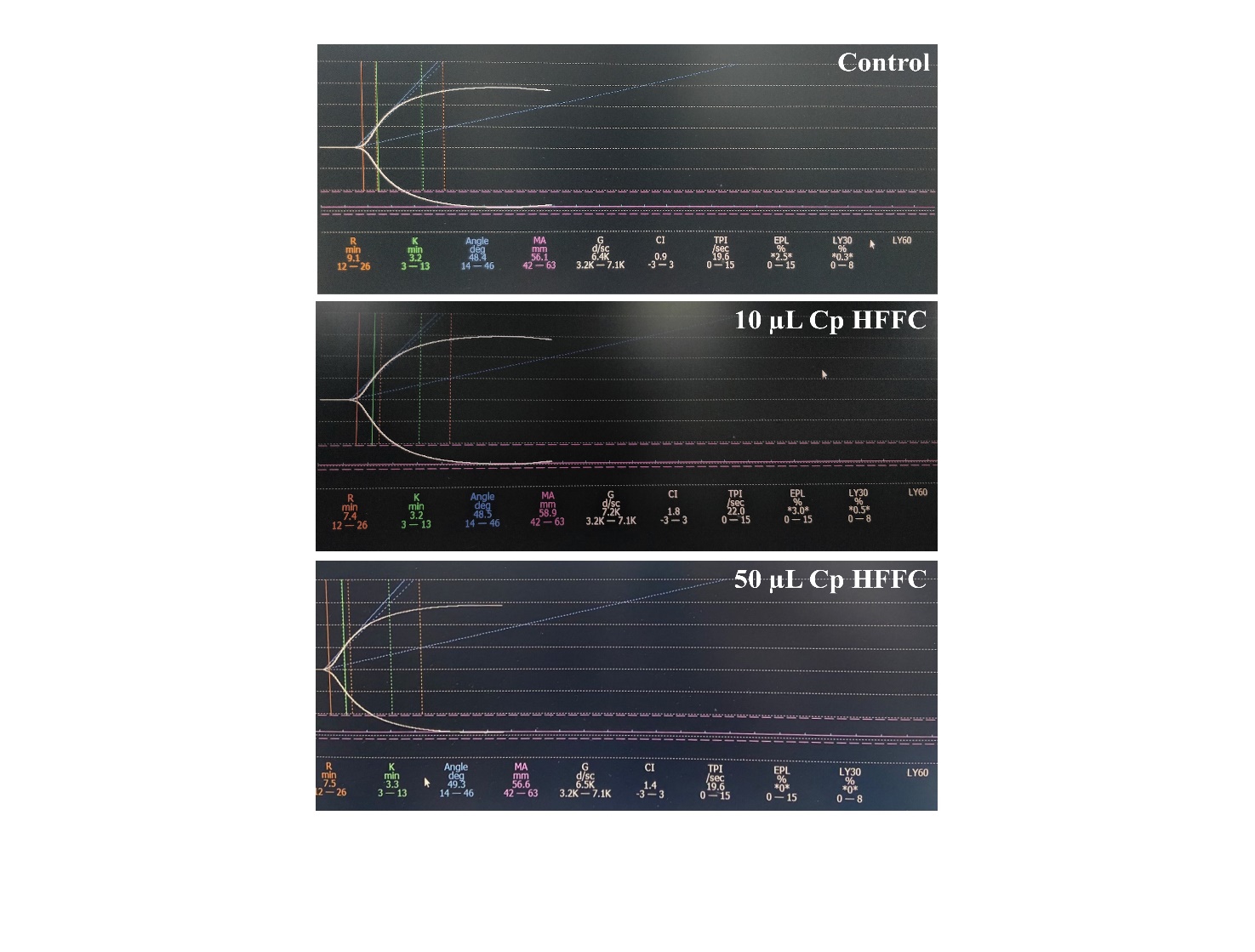


**Supplementary Figure S7.** N-TEG analysis of human blood sample, a) Represents the N-TEG graph of control (whole blood); b) Represents the N-TEG graph of Cp HFFC (10 µL) and whole blood; c) Represents the N-TEG graph of Cp HFFC (50 µL) + whole blood). All the samples (R, K, α-angle, MA, G, CI, and TPI, were estimated. R-value represents the time taken for initiation of blood coagulation, K value indicates the time needed to attain a certain level of clot strength, detected by the time taken to achieve an amplitude of 20 mm. The α- angle measures the speed at which fibrin builds, cross-linking occurs, and the rate of clot formation. MA (maximum amplitude) indicates the width of the traces representing the overall maximum attainable clot strength. An increase in tensile strength due to platelet activation and building of fibrin due to the traces increases MA or appears to widen. The clot strength (G) or firmness is calculated based on MA. G is the single most vital value of the N-TEG assay, which confirms the efficiency of the blood clot. As time progresses during the N-TEG assay, the traces at MA for a period later clot lysis commences. The clot lysis continuous for 15 min, determined by a computer algorithm. Thrombodynamic potential index (TPI) indicates the platelet kinetics index, which confirms the hypo or hypercoagulability of the blood ^6^. The R-value of the control was found to be towards hypercoagulation than the reference range of N-TEG values due to prior activation of clotting factor after blood collection from the subject. This confirms no hypocoagulability or hypercoagulability, or coagulation factors deficiency in the blood sample used for the analysis. The 10 and 50 µL Cp HFFC samples were analyzed, and the obtained values of different parameters (R, K, α- angle, MA, G, CI, and TPI) were compared with the reference ranges of N-TEG, shown in Supplementary Table S4. The reference N-TEG values of different parameters are presented in Supplementary Table S4.

Based on R, K, α angle, MA, G, and CI, we conclude that in the presence of 10 and 50 µL of Cp HFFC, quick coagulation initiation and hypercoagulation of whole blood were observed. In the presence of 10 µL of CP HFFC G, MA, CI of the clot was better compared to 50 µL and control. MA and CI were improved with minimal LY30 compared to the control. Both 10 µL and 50 µL of Cp HFFC showed blood hypercoagulation. Based on the N-TEG results 10 µL Cp HFFC is sufficient to produce or initiate the coagulation.

**Supplementary Figure S8.** The Image represents the *in vivo* blood loss in the presence of hemostatic materials in different animal models (femoral artery, liver injury, and rat tail amputation models). The data showed as mean ± SD; the error bars represent SD, n = 3, p < 0.05. Cp NFRCS has less blood loss than Ch NFRCS, and Cs treated groups. The faster the blood coagulation, the lesser the blood loss from the injury site. In all the three animal models, Cp NFRCS was able to prove its efficiency.


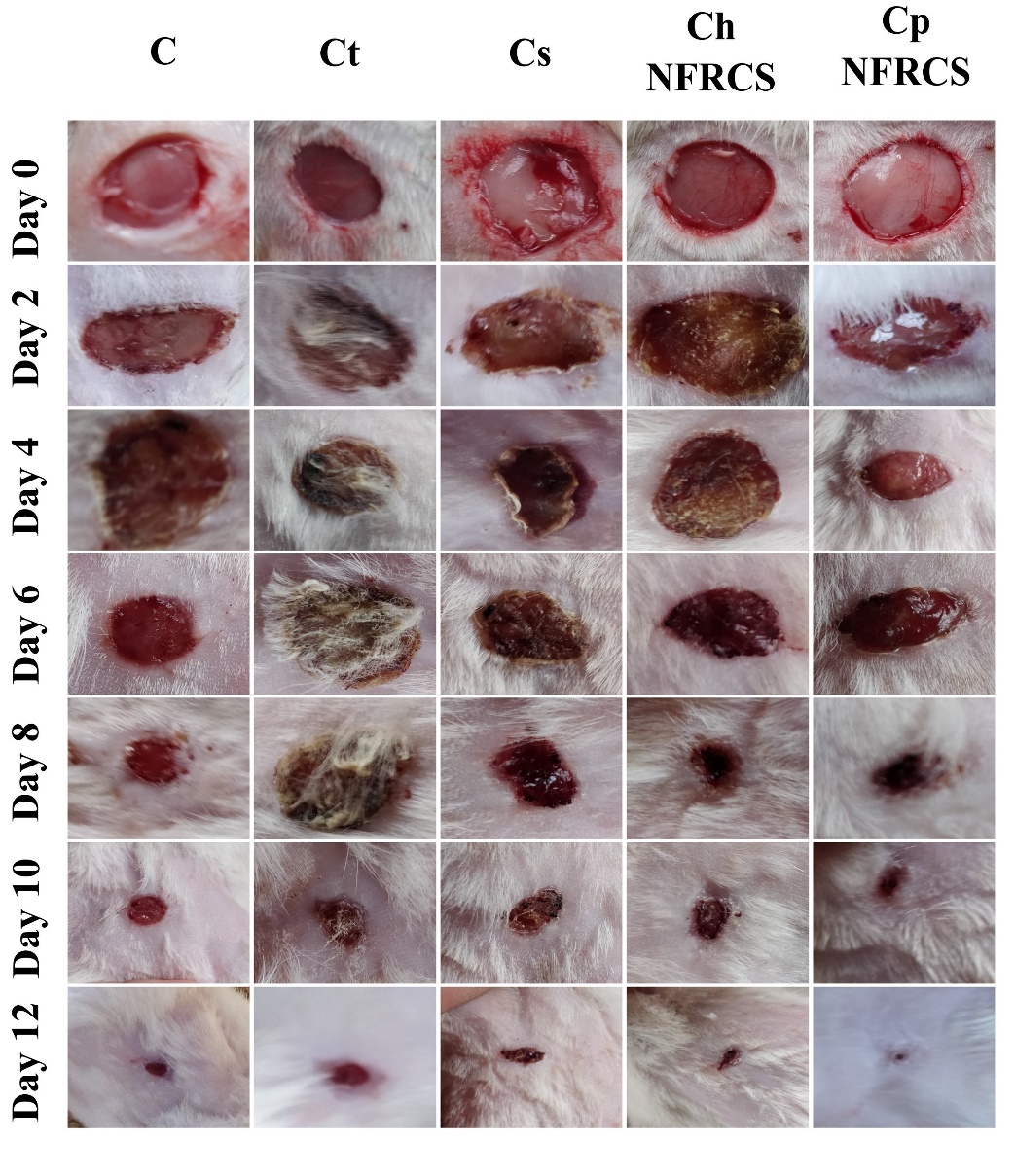
**Supplementary Figure S9.** Represents the digital images captured on every alternative day of wound healing assay (n=3). A decrease in wound area or wound closure percentage was observed in all the treated and control groups, indicating the progress of wound healing. No wound discharges or fluid was observed in the treated or untreated group. The percentage of wound healing is less in Ct treated group compared to other treatments. Ct fibers interrupted the healing process by sticking to the open wound, disturbing the wound while changing the dressing every alternative day. The groups treated with Ch NFRCS and Cp NFRCS showed a better-wound closure percentage and confirmed the uniform surface coat of the HFFC on the surface of the dispersed phase.

**Supplementary Table S5.** Uniformity of scale-up batch.

| Sample | Different portions | | | | |
| --- | --- | --- | --- | --- | --- |
|  | 1 | 2 | 3 | 4 | 5 |
| Cp NFRCS  BCT (No. of tilts) | 1 | 1 | 1 | 1 | 1 |

The scale-up batch was developed to understand whether the proposed NFRCS can be converted from bench side to bedside product. The BCT of five different portions of five different places was determined, and the BCT of all the samples was the same, confirming the uniformity of the scale-up batch of Cp NFRCS. The proposed Cp HFFC composition (continuous phase) was uniformly distributed throughout the dispersed phase.

**Supplementary Table S6.** *In vitro* characterization of Cp NFRCS stability sample

| Sample | Study | Observation |
| --- | --- | --- |
| Cp NFRCS | Color change | No color change |
|  | Folding endurance | >1000 times |
|  | BCT | 15 ± 0.00 sec |
|  | Microbial growth on the surface | No microbial growth |


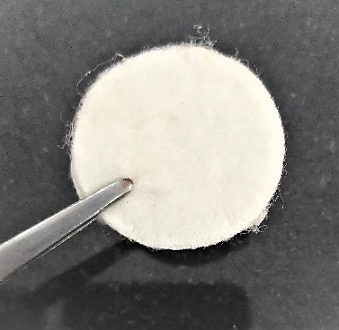
The stability of the Cp NFRCS was studied at room temperature (25℃ ± 2 ℃) for 12 months. No color change or microbial growth was observed on the surface of Cp NFRCS (Supplementary Fig. S10). The folding endurance was >1000 as the NFRCS was developed by fiber reinforcement application. The BCT was similar to the initial BCT of the fresh sample (Supplementary Table. 5).

**Supplementary Figure S10.** Digital image of Cp NFRCS of one-year stability sample.


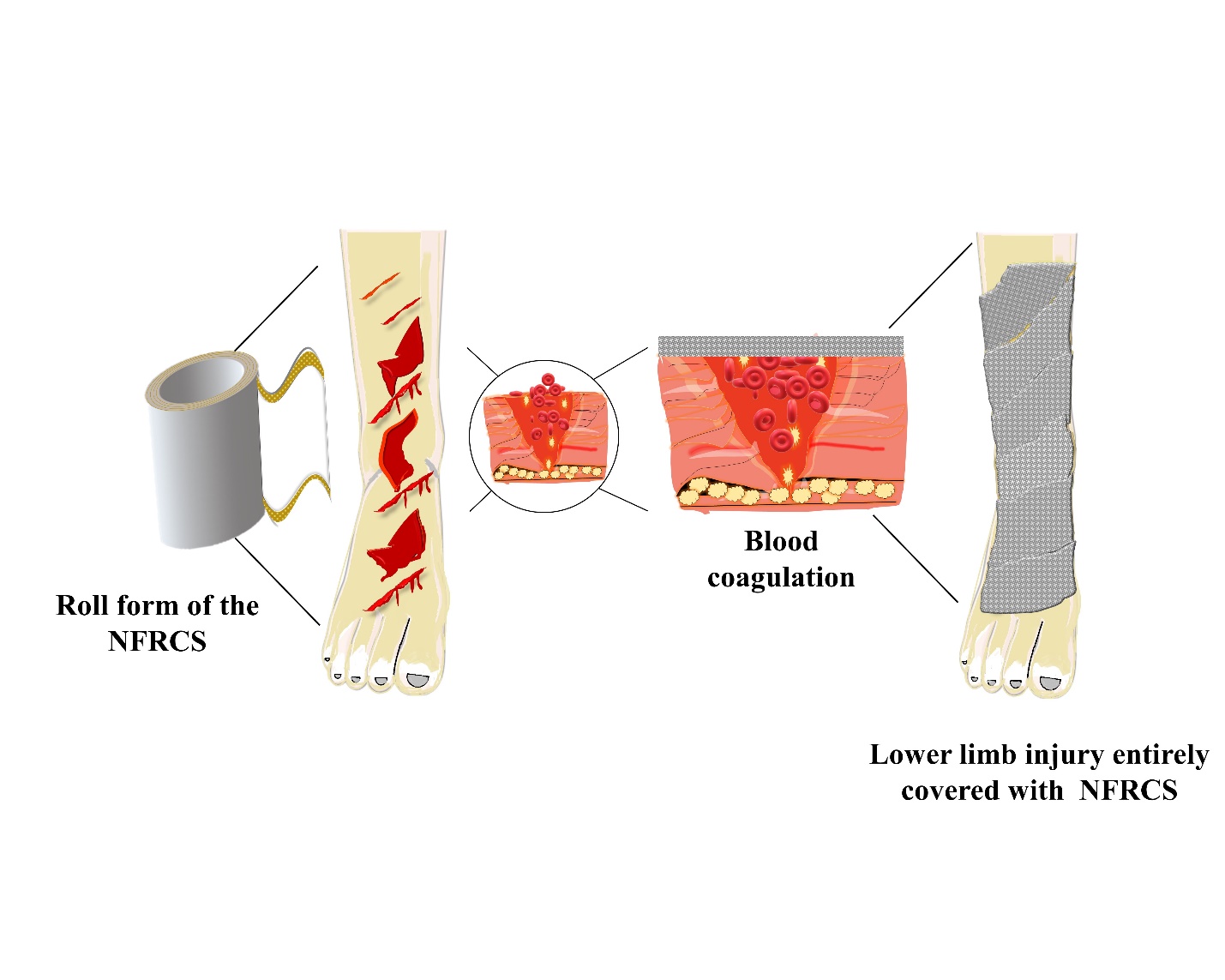
**Supplementary Figure S11.** Schematic representation of lower limb injury covered with NFRCS. The developed NFRCS readily mold as per the surface of the injury or site of application. The same NFRCS can also be used for neck injuries for which tourniquets cannot be used (pressure-sensitive injuries).

**References**

1. Durand, B. & Marchand, C. *Smart features in fibrous implantable medical devices*. *Smart Textiles and Their Applications* (Elsevier Ltd, 2016). doi:10.1016/B978-0-08-100574-3.00013-8.

2. Pina, S. *et al.* Regenerative Medicine Applications. *Materials (Basel).* **12**, 1824 (2019).

3. Li, Z. *et al.* Superhydrophobic hemostatic nanofiber composites for fast clotting and minimal adhesion. *Nat. Commun.* **10**, (2019).

4. Bessa, L. J., Fazii, P., Di Giulio, M. & Cellini, L. Bacterial isolates from infected wounds and their antibiotic susceptibility pattern: Some remarks about wound infection. *Int. Wound J.* **12**, 47–52 (2015).

5. Kalan, L. & Grice, E. A. Fungi in the wound microbiome. *Adv. Wound Care* **7**, 247–255 (2018).

6. Xuan, J., Wang, J. & Wei, B. Diagnostic Value of Thromboelastography (TEG) for the Diagnosis of Death in Infected Patients. *Clin. Appl. Thromb.* **27**, 107602962110472 (2021).
